# Considerations for designing and implementing combination HIV cure trials: findings from a qualitative in-depth interview study in the United States

**DOI:** 10.1186/s12981-021-00401-8

**Published:** 2021-10-18

**Authors:** Karine Dubé, John Kanazawa, Lynda Dee, Jeff Taylor, John A. Sauceda, Sara Gianella, Davey Smith, Steven G. Deeks, Michael J. Peluso

**Affiliations:** 1 University of North Carolina Chapel Hill, Gillings School of Global Public Health, 4108 McGavran-Greenberg Hall, Chapel Hill, NC 27599 USA; 2 AIDS Action Baltimore, 14 East Eager Street, Baltimore, MD 21202 USA; 3 Delaney AIDS Research Enterprise (DARE) Community Advisory Board (CAB), 995 Potrero Avenue, San Francisco, CA 94110 USA; 4 HIV+Aging Research Project-Palm Springs (H+ARP-PS), 1775 East Palm Canyon Drive, Suite 110-349, Palm Springs, CA 92264 USA; 5 Department of Medicine, Division of Prevention Science, Center for AIDS Prevention Studies (CAPS), University of California, San Francisco (UCSF), 550 16th Street, 3rd Floor, San Francisco, CA 94158 USA; 6 Department of Medicine, Division of Infectious Diseases and Global Public Health, University of California San Diego, 9500 Gilman Drive, La Jolla, CA 92093 USA; 7 AntiViral Research Center (AVRC), University of California at San Diego, 220 Dickinson Street, Suite A, San Diego, CA 92103 USA; 8 Department of Medicine, Division of HIV, Infectious Diseases, and Global Medicine, San Francisco General Hospital, University of California, San Francisco (UCSF), Ward 84, Building 80, San Francisco, CA 94110 USA; 9 UNC Gillings School of Global Public Health, 4108 McGavran-Greenberg Hall, Chapel Hill, NC 27516 USA

**Keywords:** HIV, HIV cure research, Combination approaches, Empirical ethics research, People living with HIV

## Abstract

**Background:**

An increasing number of HIV cure trials involve combining multiple potentially curative interventions. Until now, considerations for designing and implementing complex combination HIV cure trials have not been thoroughly considered.

**Methods:**

We used a purposive method to select key informants for our study. Informants included biomedical HIV cure researchers, regulators, policy makers, bioethicists, and community members. We used in-depth interviews to generate ethical and practical considerations to guide the design and implementation of combination HIV cure research. We analyzed the qualitative data using conventional content analysis focused on inductive reasoning.

**Results:**

We interviewed 11 biomedical researchers, 4 community members, 2 regulators, 1 policy researcher, and 1 bioethicist. Informants generated considerations for designing and implementing combination interventions towards an HIV cure, focused on ethical aspects, as well as considerations to guide trial design, benefit/risk determinations, regulatory requirements, prioritization and sequencing and timing of interventions, among others. Informants also provided considerations related to combining specific HIV cure research modalities, such as broadly neutralizing antibodies (bNAbs), cell and gene modification products, latency-reversing agents and immune-based interventions. Finally, informants provided suggestions to ensure meaningful therapeutic improvements over standard antiretroviral therapy, overcome challenges of designing combination approaches, and engage communities around combination HIV cure research.

**Conclusion:**

The increasing number of combination HIV cure trials brings with them a host of ethical and practical challenges. We hope our paper will inform meaningful stakeholder dialogue around the use of combinatorial HIV cure research approaches. To protect the public trust in HIV cure research, considerations should be periodically revisited and updated with key stakeholder input as the science continues to advance.

**Supplementary Information:**

The online version contains supplementary material available at 10.1186/s12981-021-00401-8.

## Background

The pursuit of an HIV cure remains a priority for researchers, government, community-based and funding agencies, as well as people living with HIV (PLWH) and their advocates. This is primarily due to the challenges of lifelong cumulative antiretroviral therapy (ART), including toxicities, adherence challenges, costs, and persistent HIV-related stigma [1–3]. HIV cure trials seek to either completely eliminate HIV or confer durable HIV control off ART [4]. To date, over 250 biomedical studies related to HIV cure have been conducted globally [5]. Most of these trials remain in the early stages of clinical development and carry very limited or no expectation of direct clinical benefits for trial participants [6, 7]. Some also require the interruption of ART, also known as analytical treatment interruption (ATI) [8], counter to the United States (US) Health and Human Services Guidelines for the Use of Antiretroviral Agents in Adults and Adolescents Living with HIV [8].

A growing number of HIV cure trials involve combining multiple potentially curative interventions [9]. In fact, some biomedical scientists are even asking whether single intervention in HIV cure trials should be completely abandoned because monotherapies are unlikely to lead to long-term viral suppression without ART given the complex nature of the latent viral reservoir and the compromised immune systems of PLWH [10–12]. Combination approaches will probably involve two or more clinical interventions, representing multiple mechanisms of action, with multi-modal targeting of HIV as well and immune modulating interventions to boost the immune system of PLWH [10]. Examples of combination HIV cure studies include different permutations of broadly-neutralizing antibodies (bNAbs) paired with other HIV cure modalities [13, 14], combination cell and gene modifying approaches [15], and latency-reversing agents (LRAs) combined with immune-modifying agents [11, 12, 16–19]. In the United States, there are over 20 active HIV cure clinical studies using combination approaches [5].

Combination interventions have represented significant scientific advancements in the rapidly evolving landscape in HIV therapeutics for many years, most recently the advent of US Food and Drug Administration (FDA)-approved long-acting intramuscular injectables, as well as other long-term ART in the pipeline, dosed by mouth, via transplantation and sub-cutaneous injection [20]. However, combining different HIV cure research interventions may increase clinical risks and burdens above standard ART [16, 19]. Many PLWH have nearly equivalent general population life expectancy [21]. This significantly reduces the risk thresholds that may be tolerated in these otherwise healthy volunteers [22]. Combining interventions may compound toxicities, complicate dosing and monitoring schedules, and require increased time commitments for participants accustomed to once-daily single ART tablets with limited side effects. Further, combination HIV cure trials may require agreements between regulatory agencies that oversee different products and biologics under their jurisdiction. Investigators will need to demonstrate how these complex regimens represent improvements over currently available and other pipeline HIV treatments [23].

Until now, considerations for implementing complex combination HIV cure trials have not been thoroughly considered. This qualitative study employed key informant interviews from US-based stakeholders to generate ethical, social, and practical considerations concurrently to guide the design and implementation of these trials. We elicited the perspectives of multiple stakeholder groups, including biomedical HIV cure researchers, community members, regulators, policy researchers and bioethicists on combination HIV cure research approaches under development. Through our interdisciplinary approach, we endeavored to generate considerations to ensure combination HIV cure research remains ethical and acceptable to as many stakeholders as possible—as was once the advent of combination ART (cART).

## Methods

### Study setting and participants

We used a purposive method to select key informants for our study. Our aim was to recruit a convenience sample of experts working in the field of combination HIV cure research—including biomedical HIV cure researchers, regulators, policy makers, bioethicists, and community members (e.g., PLWH or community advocates affiliated with the Martin Delaney Collaboratories Towards an HIV-1 Cure or the AIDS Clinical Trials Group). Potential informants represented academic institutions, community advisory boards (CABs), pharmaceutical companies, and regulatory agencies. We recruited participants based on their prior familiarity with the topic of combination HIV cure research. We used in-depth interviews [24] to generate considerations to guide the design and implementation of combination HIV cure research.

### Participant recruitment

The study’s principal investigator (K.D.) sent email invitations to potential informants. Email messages indicated the purpose of the study and included the institutional review board (IRB)-approved informed consent form, blank demographic sheet, and sample interview guide. We invited 20 potential informants, 19 of whom agreed to be interviewed (95% response rate). We sent interview accepters a Health Insurance Portability and Accountability Act (HIPAA)-compliant virtual conferencing weblink through which to conduct the interview. We assigned participant identification numbers consecutively following informed consent.

### Data collection

Two research team members (K.D. and J.K.) conducted all interviews in English using the IRB-approved interview guide (Table 1). Interviews took between 30–60 min to complete. Community members received an electronic US $20 gift card following their interviews; informants from academic institutions, pharmaceutical companies, and regulatory agencies did not receive compensation.

**Table 1:** IRB-approved interview guide: ethical and practical considerations for ethical and practical considerations for designing and implementing combination HIV cure trials

*Introduction* • First, thank you so much for your time Considerations for combining interventions towards HIV cure • What ethical considerations should be in place for combining different HIV cure research approaches? • What considerations should be in place when designing combination protocols? • How do we ensure combination regimens remain within acceptable benefit/risk parameters? • What regulatory considerations should be in place for combining different HIV cure research approaches? • Is it important to know the effect of each intervention when designing a combination strategy? Why/why not? Is it important to know whether combination strategies have additive/synergistic/antagonistic effects? Why/why not? • How do we prioritize which combination should be tested? What type of information is needed to make these decisions? • How many different strategies do you think we should use in combination? Can you please explain your answer? • Which one is better: administer regimens together or to administer regimens in sequence? Can you please explain your answer? • What considerations should be in place when sequencing interventions? How do we decide when to ‘time’ interventions? *Considerations for specific combination HIV cure regimens* • Do you think the following regimens should be used in combination? Why/why not? • Combination broadly neutralizing antibodies [follow-up question: what safeguards should be in place for these protocols (e.g., resistance development for one antibody?)] • Combination cell and gene modification approaches (follow-up question: what safeguards should be in place for these protocols?) • Combination latency-reversing agents with immune-modifying agents (follow-up question: what safeguards should be in place for these protocols?) *Additional considerations* • How do we ensure combination regimens represent improvements above antiretroviral therapy (ART) for people living with HIV? • What are some of the challenges of designing combination regimens? How can we best overcome these challenges? • How can we best engage people living with HIV and communities around HIV cure research involving combinations?	

### Data analysis

We audio recorded all interviews which were transcribed by a professional transcription company. One research team member (J.K.) reviewed all transcripts for accuracy against the audio recordings. We destroyed audio recordings after cross-checking transcripts for quality. Because this was a formative research project, we used conventional content analysis focused on inductive reasoning to analyze the data [24].

A research team member (J.K.) collated all de-identified responses into a single master document for manual coding. To realize the full potential of the qualitative data, we analyzed the data by question blocks. Our codebook was inductive and included code names, code descriptions, and examples. Two team members (K.D. and J.K.) double-coded the data and organized text segments into emergent themes. We then expanded and reduced themes. We resolved discrepancies by discussion and consensus during virtual meetings. The lead author (K.D.) summarized key themes and wrote narrative summaries to recontextualize the data. Co-authors subsequently reviewed the data and helped generate ethical and practical considerations to guide the design and implementation of combination HIV cure research. In addition, three informants reviewed the considerations and were extended co-authorship.

### Ethics statement

This study was IRB-approved by the University of North Carolina at Chapel Hill (UNC-CH) IRB (Study #19-0522). All interviewees provided verbal consent.

## Results

We interviewed 11 biomedical researchers, 4 community members, 2 regulators, 1 policy researcher, and 1 bioethicist. These included 11 cisgender men and 8 cisgender women, all of whom were White/Caucasian (Table 2). Interview participants worked in the field of HIV for a mean of 23.9 years (SD = 9.2 years), and in HIV cure research for a mean of 12.1 years (SD = 8.9 years).

**Table 2:** Demographic characteristics of key informant interview participants (United States, 2020–2021)

Participant number	Sex	Race/ethnicity	Informant type
01	Male	White/Caucasian	Community Member
02	Male	White/Caucasian	Biomedical Researcher
03	Female	White/Caucasian	Community Member
04	Female	White/Caucasian	Policy Researcher
05	Male	White/Caucasian	Biomedical Researcher
06	Female	White/Caucasian	Regulator
07	Female	White/Caucasian	Biomedical Researcher
08	Female	White/Caucasian	Regulator
09	Male	White/Caucasian	Biomedical Researcher
10	Female	White/Caucasian	Biomedical Researcher
11	Male	White/Caucasian	Biomedical Researcher
12	Female	White/Caucasian	Biomedical Researcher
13	Male	White/Caucasian	Biomedical Researcher
14	Male	White/Caucasian	Bioethicist
15	Male	White/Caucasian	Community Member
16	Male	White/Caucasian	Biomedical Researcher
17	Male	White/Caucasian	Community Member
18	Male	White/Caucasian	Biomedical Researcher
19	Female	White/Caucasian	Biomedical Researcher

The summary of ethical and practical considerations for designing and implementing combination HIV cure trials can be found in Table 3.

**Table 3:** Summary of ethical and practical considerations for designing and implementing combination HIV cure trials

*Considerations for combining interventions towards an HIV cure* • Ethical considerations for designing combination HIV cure research regimens—as for other types of trials—include a strong scientific rationale, social value through prioritization and optimization, balancing efficacy and safety, risk minimization and mitigation, and robust informed consent • Trial design considerations include striking a balance between pursuing novel scientific paradigms and repurposing already existing products, ensuring careful statistical design for signal detection, maximizing potential efficacy while minimizing safety concerns, carefully selecting trial participants through clear inclusion/exclusion criteria, minimizing participant burdens such as the number of study visits/procedures, and planning for long-term follow-up of participants • Considerations for ensuring an acceptable benefit/risk balance for combination HIV cure regimens include having robust pre-clinical safety data about single interventions, minimizing risks for otherwise healthy volunteers, maximizing the likelihood of scientific benefits, and ensuring consensus in the scientific community that a combination is worth testing • Regulatory considerations for combination HIV cure trials include consulting existing FDA Combination Products Guidance Documents (https://www.fda.gov/regulatory-information/search-fda-guidance-documents/combination-products-guidance-documents), collaborative regulatory reviews and case-by-case analyses, relying on the pre-IND consultation process, higher scrutiny for combination trials compared with monotherapies, evidence for intended proximal biological effect, clear endpoints, hearing the patient voice and meeting unmet medical needs • From a regulatory and safety standpoint, it is important to know the effect of each intervention before testing them in combination. Knowing whether interventions have additive versus synergistic efficacy may be less important than excluding for antagonism or synergistic toxicity • Focused attention should be given to prioritizing combination HIV cure strategies given limited resources and eligible trial participants. Considerations for prioritizing combination HIV cure research approaches included ensuring the “greatest positive results with least danger,” complementary mechanisms of action, future clinical viability, and patient friendliness • The optimal number of interventions to include in combination HIV cure trials will depend on the interventions being tested. The number of interventions ultimately affects toxicity, complexity, interpretability, practicality, and scalability • Decisions whether to administer interventions together or sequentially depend on the agents being tested, as well as their mechanisms of action, routes of administration, and side effects. Sequential administration may be warranted to measure the effect of each intervention and to prevent overreactions. Concurrent administration may be required if agents are targeting different issues or preventing resistance. The simpler the sequence, the higher the likelihood of future uptake and adherence in the real-world. The optimal timing of interventions will also depend on the mechanism of action. Biomedical researchers should rely on robust PK/PD and modeling data to determine optimal timing or dosing windows. The cancer field may provide a precedent and guidance *Considerations for specific combination HIV cure regimens* • bNAbs are viable candidates for combination HIV cure regimens, either with each other or with other HIV cure research modalities, due to their high safety profile, good characterization, and the fact that they are human-derived. However, bNAbs do carry some clinical risks (e.g., immune complexes, allergies) that should be monitored. Considerations should also be given to bNAb costs, scalability and accessibility. Safeguards should be in place to reduce resistance development, caused, for instance, by similar decay profiles that will result in functional monotherapy • Combining cell and gene modification approaches warrants caution because of the potential for immediate and future irreversible conditions and serious and life-threatening adverse events. Possible safeguards include close FDA and Institutional Biosafety Committee oversight, following stepwise approaches, testing for off-target effects, staggering interventions and trial participants, clear stopping rules in case of intolerable toxicity of futility, and long-term follow-up of trial participants • There may be a strong scientific rationale for combining LRAs and immune-based agents. Safeguards should include ART maintenance during LRA-only studies, long-term follow-up of trial participants, and community involvement in designing combinations • Several types of novel combination regimens may emerge in the future. We will need to continue ensuring these strategies remain within acceptable benefit/risk parameters for otherwise healthy PLWH *Additional considerations* • To ensure combination HIV cure regimens represent improvements for PLWH, there needs to be a clear definition of the projected clinical benefits. This may require significant input from PLWH. Issues of accessibility, affordability, and stigma also need to be considered • Designing and implementing combination HIV cure regimens may present additional challenges, such as IP issues and pharmaceutical company cross-collaborations. Incentives should be provided and public–private partnerships created to promote company collaborations. Researchers should continue working on assays and biomarkers that can identify and predict viral rebound • Community and societal aspects of combination HIV cure research should also be carefully considered, such as managing expectations and building research literacy. Combination HIV cure regimens should also be designed with the end product in mind for eventual equitable real-world implementation	

### Considerations for combining interventions towards HIV cure

We explored considerations related to: (1) ethics of combination HIV cure research interventions, (2) trial design, (3) acceptable benefit/risk profiles, (4) regulatory considerations, (5) determining effects of interventions, (6) prioritizing combinations, (7) optimal number of interventions, and (8) sequencing and timing of interventions.

#### Ethical considerations for combination HIV cure

We asked informants to provide ethical considerations for combination HIV cure regimens. Several informants expressed belief that there must be a strong scientific rationale and a solid evidence base for moving combinations forward into human testing. To ensure social value, combinations will need to be prioritized on the basis of potential efficacy. Attention should also be given to minimizing and mitigating risks, optimizing combinations, understanding drug-drug interactions, and ensuring participants’ informed consent.

Two regulators (#06, #08), a policy researcher (#04), and three biomedical researchers (#02, #05, #18) believed combination approaches will be required for durable ART-free control of HIV.

*I think everybody agrees from a scientific perspective that almost certainly we're going to have to have a combination of approaches*.—Biomedical Researcher (#02)

To ensure social value, informants recommended carefully prioritizing combinations for clinical testing, discussed further below. According to one biomedical researcher (#02), prioritization would preserve precious resources and reduce opportunity costs for participants. The bioethicist (#14), however, deplored the lack of effective techniques for prioritizing combinations because intellectual property (IP) considerations may sometimes take precedence over patient welfare.

Informants converged on the need to balance efficacy and safety when designing combinations. Most informants recognized that combination regimens would elevate the clinical risks above the standard of care and that ethicality will depend on researchers’ ability to minimize and mitigate overall risks. A policy researcher (#04) and two biomedical researchers (#11, #19) advised ensuring each component is safe before testing in combination.

*I guess you would want to really first know that step A is relatively safe on its own, and then that step B is relatively safe on its own, and really look very, very deep into what could possibly go wrong if you sequentially use A and B… For combinations, it's a multi-step process that needs to really be in place. We always talk about minimizing the risk and maximizing the potential benefit to whatever extent, but I think at this stage really minimizing the risk is the most important part.*—Policy Researcher (#04)

Four informants (#03, #04, #09, #11) identified the need to understand drug-drug interaction profiles when designing combinations. Similarly, the bioethicist (#14) and a biomedical researcher (#11) described the ethical duty to optimize the combination regimen based on critical variables, such as safety and efficacy.

*Whenever you have a therapeutic, it has to be optimized… When you're doing a combination, you've got two axes that you have to optimize... One sort of anxiety I have is that there is more probability of missing the optimum and carrying a candidate forward from phase I into phase II that may be more toxic than needed, or that may be not quite as effective as it could be.*—Bioethicist (#14)

*How do you find the optimal dose of two things? It's obviously more complicated than finding the optimal dose of one thing. Finding the optimal timing of two things is different than the optimal timing of one thing.*—Biomedical Researcher (#11)

Additional ethical considerations included the need to test combinations that PLWH would be willing to tolerate (Policy Researcher, #04) and robust participant informed consent around the potential for increased known and unknown risks when compared with monotherapies (Bioethicist, #14 and Regulator, #08). A regulator (#06) stated that most combination HIV cure trials remain in the experimental medicine stage.

#### Trial design considerations for combination HIV cure

Informants provided considerations for designing combination HIV cure trials. Although not unique to combination trials, these included maximizing both safety and efficacy information, carefully selecting trial participants, minimizing participant burdens, and ensuring long-term follow-up of trial participants. One community member recommended including combinations in the trial design that could be implementable in the real-world, i.e. that are scalable and cost-permissive for use in low- and middle-income countries (LMICs).

The bioethicist (#14) questioned the trade-off between investigating novel scientific modalities versus repurposing older drugs. This bioethicist expressed the worry that biomedical scientists often do “research of opportunity” with currently available products that have a lower chance of success instead of pursuing novel drug development. Further, the bioethicist (#14) suggested paying attention to statistical design to be able to detect a signal if there is one.

*The question that I sort of struggle with at a philosophical but also empirical level is: what's the optimal trade-off between repurposing drugs that are already on the shelf by combining them in different ways versus investing in completely new drugs…? I suspect we have not figured out the right optimum… I do worry… that a lot of the clinical research we do is research of opportunity as opposed to research that has a good prospect of actually impacting [the] disease, particularly in the combination realm.*—Bioethicist (#14)

*I think that it's easy, and dangerously easy, for researchers to throw a lot of different drugs and hypotheses into a single phase I study without doing the proper statistical design to maximize the prospect of detecting signal.*—Bioethicist (#14)

Further, informants advised selecting combinations that show great potential for synergistic efficacy while minimizing overall safety concerns. A regulator (#08) recommended designing trials to maximize both safety and efficacy information that could be derived from the trial.

Additional trial design considerations related to trial participant selection. Researchers must be rigorous with their inclusion/exclusion criteria to avoid risk of undue harm. Further, a policy researcher (#04) and a regulator (#08) advised starting with a small number of trial participants and building in as many safety parameters as possible in early-phase combination trials.

*Really think about that [selection of trial participants] very carefully and, as always, just start with very, very small patient numbers and learn as much as you can from each individual patient.*—Policy Researcher (#04)

*The trial design is very important, that as much safety parameters can be built in. For example, you might consider moving slowly with the trial, having one or two participants receive the intervention before you go on to using the intervention in a lot of other patients or trial participants.*—Regulator (#08)

The bioethicist (#14), a biomedical researcher (#09), and a community member (#15) recommended trying to minimize burdens on clinical trial participants (e.g., study visits, procedures), because combination trials can be more demanding than monotherapy trials.

*Another part is related to not only the burden on the participant of dose administration, where they have to go to have this procedure done, et cetera, is the sampling, and what kind of follow-up, et cetera… But what we're talking about is how many visits, how many times am I going to be stuck or prodded, or what compartments, what kind of samples, and how often. I think trying to minimize the burden of the patient in participating in these studies is really important because you are asking a lot.*—Biomedical Researcher (#09)

A biomedical researcher (#05) and a community member (#03) recommended planning for long-term monitoring of trial participants because some HIV cure research interventions can have prolonged effects or potential adverse events, such as cancer that do not develop until many years after trial completion.

Finally, a community member (#15) expressed the need for combination trials to be designed to yield clear scientific answers. Combination HIV cure trials should also be designed with end products in mind.

*Just kind of throwing a lot of things at people and then not having the statistical power to sort of figure out whether it's contributing to any kind of beneficial outcome, I think it's problematic. I think part of ethics is ensuring that people are participating in a study that's going to be able to answer a question, even if it's not the answer everybody wants.*—Community member (#15)

*It’s a bit of a bigger picture thing: is the effect that you're hoping to get something that eventually could be translated possibly into something simpler if technology improves, or are you just testing an incredibly complicated thing that would always be incredibly complicated even if it worked? So, just thinking about what the end goal is,… if you see what you're hoping to see.*—Community member (#15)

#### Ensuring acceptable benefit/risk profiles

To ensure acceptable benefit/risk profiles for moving combination HIV cure regimens into human testing, informants noted the need for robust pre-clinical safety data as for any trial. Further, the fact that PLWH on ART are now considered otherwise healthy volunteers reduces the risk threshold for what can ethically be tolerated. There should also be general agreement in the scientific community that the benefit/risk profile is acceptable before testing specific combinations.

Although not unique to combination trials, a bioethicist (#14), regulator (#06), biomedical researcher (#09), and community member (#15) suggested extensive pre-clinical safety data before moving combinations into human testing. They also noted that the safety bar is much higher for interventions that have never been tested in humans. A regulator (#08) advised that non-human primates may be better able to predict what happens in humans than humanized mice.

Further, a regulator (#08) recommended understanding the risks posed by individual interventions within the framework of the target patient population. In this case, PLWH were recognized as otherwise healthy volunteers given the potency of current ART, and this reduces the amount of risk that can ethically be tolerated. The bioethicist (#14) echoed this opinion and noted PLWH may have a lot to lose from early-phase studies.

*I think it starts with understanding the risks of the individual intervention [with]in the framework of the patient population. The challenging aspect of a lot of HIV cure research is that most patients who are really, really good clinical trial participants, in other words they adhere to their medications already, they're able to come to study visits, they're able to swallow their medications, et cetera, those patients are often doing quite well on existing therapies and might not have many adverse events with the therapies that they're receiving.*—Regulator (#08)

*I think there's often an assumption that patients have nothing to lose by going into a phase I study. And I think it's a very deeply problematic way to understand early phase studies. Patients have lots to lose from participating.*—Bioethicist (#14)

To ensure benefit/risk balance, a biomedical researcher (#18) advised maximizing the likelihood that something would be learned scientifically from the trial (scientific benefits), while minimizing potential risks to trial participants. A second biomedical researcher (#12) noted participants may derive tremendous though immeasurable altruistic or psychosocial benefits from participating in trials, thus tipping the benefit/risk balance. A third biomedical researcher (#10) noted that there should be general agreement in the scientific community that the benefit/risk balance is acceptable before moving forward with testing specific combinations.

*There needs to be… a consensus: this is the amount of risk we as a community of investigators, and community representatives, and regulators are able to handle.*—Biomedical Researcher (#10)

#### Regulatory considerations for combination HIV cure

Informants also provided regulatory considerations for combination HIV cure trials. Several informants pointed to the U.S. FDA’s existing guidance for combination trials and the fact that combination protocols would require collaborative reviews across agencies. Regulators advised consulting with the FDA early and often when preparing combination trial applications.

A regulator (#06) explained that regulation is as much about science as it is about ethics. Likewise, a policy-researcher (#04) viewed regulation as making careful judgments about benefits and risk, as well as accounting for uncertainty. The policy-researcher (#04) discussed the critical importance of hearing the voice of the patient as part of the regulatory process.

Several informants pointed to the established FDA guidance on combination trials. However, a biomedical researcher (#09) said the guidance was more explicit for existing products than for novel investigational products.

*There are regulatory guidance documents for developing combination products, but they do have gaps because some of them only speak of agents that exist already versus novel agents. And so, there's probably some more work to do with the agency.*—Biomedical Researcher (#09)

Further, a biomedical researcher (#19) described the FDA as very supportive and attuned to the science of combination HIV cure research. A biomedical researcher (#09) and two community members (#03, #15) praised the FDA for doing thoughtful collaborative case-by-case reviews of combination HIV cure protocols, particularly across the Center for Drug Evaluation and Research (CDER) and the Center for Biologics Evaluation and Research (CBER).

*My personal experience with the FDA… is that they are very in-tune with the science behind cure research, and they're open to supporting these combination strategies to move forward.*—Biomedical Researcher (#09)

*I think it probably needs some collaboration with FDA and I think there are dialogues between CDER, the drug people, and CBER people... And so I think it's important that when they get protocols that involve combining different categories, they have some mechanism for doing collaborative regulatory review.*—Community Member (#15)

Two regulators (#06, #08) advised biomedical researchers to rely on the pre-Investigational New Drug (IND) consultation process and confer with regulatory authorities early and often when designing combination trials. A biomedical researcher (#13) expressed that there should be heightened regulatory scrutiny around combination trials compared with monotherapy trials due to the potential for synergistic toxicity.

Moreover, two biomedical researchers (#12 #19) advised collecting robust safety data in humans for each monotherapy before moving to combinations. Another biomedical researcher (#02) suggested having reasonable evidence that each agent has the “intended proximal biological effect” before proceeding to combinations. This same biomedical researcher (#02) further warned that sponsors may be reluctant to have agents tested in combinations because any adverse events may significantly set back all agents in the combination.

*There isn't actually any rule that prevents you from combining two investigational agents, the challenge is always that the sponsors of both agents have to be willing to have them combine. Especially early on when there may not be much known about each agent on its own and one sponsor typically is willing to be tarred by the brush of the other sponsor if there's an adverse event that may in reality be due to the other intervention, but both interventions get labeled with the serious adverse events.*—Biomedical Researcher (#02)

In turn, the bioethicist (#14) observed that, compared with the FDA, individual IRBs often lack the requisite experience necessary to review early-phase combination trials which could be very problematic. The bioethicist (#14) noted that later-phase combination trials are easier for IRBs to review due to the cumulative body of scientific evidence around safety.

*My experience with serving on [institutional] review committees is they don't really look at these data. They just take investigator's word for it that this is viable and/or maybe they work in the field and they know if this is a really good promising candidate. But I don't think people really scrutinized those data very carefully... My sense is they're pretty reluctant to say no to phase I studies… With phase III trials or later phase trials, it's easy to be systematic and transparent; you can always do a systematic review and you see your point estimate and the confidence intervals exclude or contain some effect that you want to look for, and you can say, "Yeah, we know this is a good risk benefit balance, and we know this is a viable hypothesis, because this is the totality of the evidence."*—Bioethicist (#14)

A major issue complicating regulatory reviews of combination HIV cure trials is the absence of a clear endpoint for HIV cure research. This issue remains a “long-standing debate” in the field, as noted by a biomedical researcher (#09), because viral load is the only currently accepted endpoint.

*Well, clinical endpoints and plasma viral load are they only things that you can get an indication for an HIV medicine. So, how do you navigate an endpoint that isn't based on plasma viral load, especially in a healthy population where you're not anticipating clinical endpoints? … What's your endpoint for showing you've actually done something?*—Biomedical Researcher (#09)

In addition to defining a clear endpoint, there should be a clear unmet medical need for the patient population.

*I think any regulator's first question would be: what is the unmet medical need? If they're not convinced that there's an unmet medical need, then of course they're going to think very, very conservatively about this… If the regulator believes that there is an unmet medical need…then they will be more willing to really look at this in terms of what can be done.*—Policy Researcher (#04)

Finally, a bioethicist (#14) and policy researcher (#04) converged on the precautionary principle, the principle that the introduction of a new product or process whose ultimate effects are disputed or unknown should be resisted. They advised regulators must remain risk averse when evaluating combinations to preserve faith in the entire HIV cure research enterprise.

#### Determining individual and combination effects of interventions

We asked informants about the importance of knowing the effects of single interventions before designing combination regimens. All informants except a biomedical researcher (#16) said it was important to first know the effect of single interventions before combining them. Knowing whether interventions had synergistic versus additive effects was perceived as less critical. Informants recommended excluding combinations that may be antagonistic.

Most informants indicated that it was important to know the effect of individual components before combining them. This was perceived to be critical from a regulatory and safety standpoint.

*From a regulatory point of view… obviously you need to know that each piece of the combination is having an effect. You can't just have something in there and not know what's contributing to the positive effect because then you're only getting potential risks.*—Regulator (#06)

However, one biomedical researcher (#16) diverged in their opinion and stated that knowing the effect of each intervention was not critical if the overall combination regimen worked.

*I don't necessarily think, as a clinical trialist or as a clinician, [we] need to know how each one works by itself. It's important, though, to have some evidence that both are needed… I don't think you need to know how things work by themselves to do the initial studies but, ultimately, you need to be able to prove that everything that you're doing is necessary. So, that may require going back later and subtracting things one by one.*—Biomedical Researcher (#16)

Several biomedical researchers expressed the ideal combination for HIV control would have either an additive or a synergistic effect, akin to cART. However, the difference between additive versus synergistic effects was perceived as less critical to ascertain, except in the case of synergistic or overlapping toxicities.

*I mean, you could argue like ART, we use the synergistic effect of the individual drugs in order to prevent resistance, and similarly, I think that is something that should be done and should be studied, and I would assume that in most studies that would be part of the aims and readouts*.—Biomedical Researcher (#13)

*To my mind, it doesn't really matter a whole lot whether interventions are merely additive or are synergistic because in part that becomes a bit of an academic exercise.*—Biomedical Researcher (#02)

Three biomedical researchers (#02, #09, #12) recommended excluding combinations that may be antagonistic.

#### Prioritizing combinations to be tested in humans

Informants provided considerations for prioritizing combination HIV cure regimens to be tested in humans. Combination concepts with the strongest scientific rationale and “the ones that seem most likely to yield positive results with the least danger” (Community Member, #01) were considered a priority. Biomedical researchers recommended prioritizing combinations that target multiple regions of the immune system, as opposed to overlapping pathways.

Most informants acknowledged the need to prioritize combination HIV cure regimens. Strategies that would show complementary mechanisms of action or that would elicit multiple arms of the immune system should be prioritized. In turn, strategies that would engage similar pathways should be deprioritized.

*But in terms of what we would have as the top priorities… by specific mechanisms of action where we think that the activity of the one drug is going to unlock the ability of the other drug to do what it's supposed to do… I guess that the other thing that I would potentially add on that is, if we're looking at things, in particular from a remission standpoint, bringing in multiple arms of the immune system makes a lot of sense… So, combinations that elicit an antibody response and also a T-cell response, that's kind of a fundamental example, are probably things that should go to the front of the line because …[of] the lessons we've learned from antiretroviral therapy that the virus is going to try to escape in any direction that it can, so if you have combinations that are going to block those escape pathways, those I think should be front of the line.*—Biomedical Researcher (#11)

*I guess to give a counter example [from the field of latency-reversing agents] … [we should] probably not [prioritize] two things that are going to go after the same pathway.—*Biomedical Researcher (#11)

Another biomedical researcher (#09) recommended prioritizing combination regimens that are most clinically viable and patient friendly.

*I think you kind of have to demonstrate first that you have a viable clinical candidate that would be as patient-friendly as it can be, and then with that, that's when you start looking at which ones are ready for prime time, if you will, in humans.*—Biomedical Researcher (#09)

Finally, a biomedical researcher (#13) described the lack of opportunities to debate priority combination HIV cure products. Another biomedical researcher (#19) explained that prioritization of combinations remains critical for the HIV cure research field to avoid scientific overlaps and “me too” combinations.

#### Determining the number of interventions to be included in combination HIV cure regimens

We asked informants to provide considerations for determining the optimal number of interventions to be included in combination HIV cure regimens. There was no convergence on the specific number of desired interventions. However, most informants noted that a small number of interventions would be preferable for practicality, toxicity, interpretability, and scalability reasons. Most informants advised proceeding in a stepwise fashion given the “double-edged sword” nature of combination regimens. The bioethicist (#14) advised exploring adaptive protocol designs that would provide more flexibility to add or subtract interventions, particularly in later-phase trials.

Informants explained that the number of interventions in a combinatorial HIV cure regimen would depend on the specific interventions being tested. They noted, however, that a smaller number of interventions would be preferable for practicality and scalability reasons.

*I don't know that it's easy to come up empirically what some upper bound should be. I think, certainly, the smallest number of interventions that can be combined the better. We certainly have seen from antiretroviral therapy that mega-HAART [Highly Active Antiretroviral Therapy] wasn't a solution… But I think we do have to be mindful of what is ultimately going to be practical. I would have some skepticism at an intervention that requires four or five different modalities.*—Biomedical Researcher (#02)

Several informants advised proceeding in a stepwise fashion before throwing “the kitchen sink” at HIV (Community member #01).

*Well, I think you do it in a stepwise manner, right? Because… when you start combining things, you don't know how they're going to interact in the body, and the immune system is a very complex system. So…, you don't start with the kitchen sink… When in doubt, start small and work your way up.*—Community Member (#01)

A biomedical researcher (#11) noted the “double-edged sword” nature of combination HIV cure trials. While combination ART required three drugs and current ART is efficacious with two drugs, there is no guarantee this will be the case for durable ART-free virologic control. However, HIV cure science is in its infancy, and some informants expressed skepticism over combining more than three interventions, but this also depends on the interventions being tested.

*The more agents you add, it probably increases the complexity of the system exponentially, but also the opportunities of the system exponentially. It's a double-edged sword; … I think starting with two or three agents in a combination feels to me more manageable, but maybe people who've been doing this for a lot longer time feel more comfortable… For antiretroviral therapy, the magic number was three. Who knows what we're looking at in the future here?*—Biomedical Researcher (#11)

Four biomedical researchers (#05, #10, #13, and #16) did not think there should be an upper limit on the number of interventions. A biomedical researcher (#16) noted the issue was less about the number of interventions, and more about overall toxicity, complexity, efficacy, and interpretability.

*The number of drugs is not really so much the issue as how complicated the regimen is and what kind of side effects it might have… The real issues are toxicity and complexity and efficacy, which may or may not have anything to do with the number of drugs.*—Biomedical Researcher (#16)

A regulator (#06) cautioned against combining too many products in a single regimen because any untoward event may result in discarding all agents in the combination.

*It also becomes a problem if you get an adverse event. You have to kind of throw out the whole combination and you don't even know if you've got some sort of preliminary measure of potency of any one agent. You have to be able to tie your first event to what it is; otherwise, you're going to end up throwing everything out and it makes it that much harder to study the remaining components.*—Regulator (#06)

The bioethicist (#14) advised exploring novel adaptive clinical trial designs that provide more flexibility by allowing investigators to add or subtract agents to an already existing protocol platform—particularly in later-phase trials. These trial designs have been used in oncology and coronavirus disease 19 (COVID-19) and should also be explored in the HIV cure research field.

*It's called the keynote trial but, in principle, you can statistically design a trial so that you can have the never ending set of combinations that are just flowing into and out of it, like a master protocol… But in many ways, it would be great if we had more kind of master protocol mentality where we had just basically a machine that we plug in just one protocol and combinations are flowing in and flowing out in an adaptive way.*—Bioethicist (#14)

#### Considerations for sequencing and timing of interventions

Informants noted that considerations for administering interventions concurrently or sequentially will depend on the interventions being tested, including mechanisms of action and routes of administration. Sequential administration would allow for more precise measurements around each intervention. Concurrent administration should be considered when multiple interventions are required simultaneously to achieve the desired activity.

Informants noted that the order of administration will be dependent upon the interventions being investigated. A community member (#15) recommended considering potential side effects as well.

*I think it depends on the science behind what we think is going to be the most effective approach to get to a cure… There may be a real biological impact of the order in which those are given.*—Biomedical Researcher (#05)

*I think it depends on what you're trying to do and then what the options are. I think the science dictates how you administer whatever the therapy or treatment is.*—Community Member (#01)

Regulators (#06, #08) and biomedical researchers (#02, #11) described how sequential administration would permit more precise measurements around each intervention. Examples of interventions that should be administered in sequence included immune-based approaches such as therapeutic vaccines or vaccines followed by antibodies, either to enhance effects or prevent overreactions.

Informants also advised that interventions will eventually need to be translatable and implementable in the real-world. In this respect, concurrent administration was believed to increase eventual uptake and adherence while minimizing burden.

*You look at what's going to happen in the real world, right? I mean, in the context of a clinical trial, you can do almost anything with a highly motivated volunteer... If that's what the treatment ends up being, is it going to be feasible and will people actually follow through and get it, or can they take that much time off work to keep going in every week for a series of immunizations or whatever it takes or monoclonal antibody infusions or whatever?*—Community Member (#01)

We also asked informants to provide considerations for timing interventions. Once again, informants converged on the notion that the mechanism of action would dictate the timing of interventions. When testing interventions in combinations, scientists would not want to miss unique “window[s] of opportunity” (Biomedical Researcher, #11).

Biomedical researchers advised relying on pharmacodynamic (PD)/pharmacokinetic (PK) data to determine optimal timing of interventions. For LRAs and checkpoint inhibitors, timing may be a matter of hours or days depending on cycles of viral blips or expression. For immune-based approaches, timing will likely be a matter of weeks or months depending on the immune response.

### Considerations for specific combination HIV cure regimens

We explored considerations related to combining: (1) bNAbs, (2) cell and gene modification products, and 3) LRAs and immune-based interventions.

#### Combining broadly neutralizing antibodies

Informants provided overwhelming support for using bNAbs, molecules that can neutralize multiple HIV strains, in combination regimens with each other or other HIV cure research modalities. They commented on the excellent safety profiles of bNAbs and their promising use as viable components of future combination HIV treatment or cure regimens. Some issues were raised around bNAb costs, scalability and accessibility. Safeguards for using bNAbs included pre-screening for sensitivity, protecting against resistance, monitoring risks, and surveilling viral populations.

Most informants stated bNAbs should be used in combination regimens due to their proven safety profile. However, a noted risk of bNAbs is the creation of immune complexes, also called antigen–antibody complexes, that induce inflammation (Biomedical Researcher, #12).

Further, bNAbs were portrayed as good candidates for combination regimens because they have been well-characterized and are human-derived. Informants recognized them as promising components of future combination HIV cure regimens, or as the “backbone of a combination strategy” (Biomedical Researcher, #10).

Biomedical researchers further commented on recent scientific developments to improve the potency, breadth, and delivery mechanisms of bNAbs (Biomedical researcher, #05). However, they raised concerns around the timing of infusions, high costs, and difficulty of access. A biomedical researcher (#18) did not believe intermittent bNAb administration represented a “functional cure” for HIV.

*Giving people bNAbs with a half-life of six months for the rest of their life, is not a functional cure. That's another antiviral.*—Biomedical Researcher (#18)

In terms of possible safeguards for using or pairing bNAbs, biomedical researchers mentioned the need for sensitivity screening and safeguarding against potential resistance. Possible ways to lessen risk of resistance included using bNAbs in combination, prioritizing combinations to prevent escape mutations which cause viral replication, resulting in resistance, using bNAbs with similar half-lives to reduce likelihood of monotherapy, and shortening the length of the ATI.

A biomedical researcher (#10) and a community member (#15) advised including the risk of bNAb resistance as part of the informed consent process. Additional safeguards for combining bNAbs included monitoring risks of allergies and continuous surveillance of the viral population (Policy Researcher, #04).

#### Combining cell and gene modification products

Compared with bNAbs, informants expressed more caution with combining cell and gene modification approaches, either with each other or other HIV cure research modalities. They also issued several safeguards, such as ensuring product specificity, testing for off-target effects, and mandatorily following trial participants long-term after a trial has ended.

Informants expressed caution when combining cell and gene modification products due to their potential for high clinical risks, irreversibility, and long-term side effects. The type of cells being modified (e.g., stem cells versus mature cells) also needs to be considered (Biomedical Researcher, #02).

*Well, here you get into a bit more risky territory because obviously the gene editing. The principal concerns are whether you are having off target effects and could there be some kind of risk for oncogenic transformation of the cells.*—Biomedical Researcher (#02)

*It's not like a pill that you can take and… you get this adverse event and then it's gone when it's out of your system. I mean, once you start zinc fingering and scissoring around, and then you've done it.*—Community Member (#03)

Some informants valued the scientific rationale for pairing cell and gene modification products, given that the first two cases of HIV cure—Timothy Brown and Adam Castillejo—involved some form of gene modification. Regulators were receptive to cell and gene combination products but stated these would require close oversight (Regulator, #08). The target population would also need to be considered (Biomedical Researcher, #11).

*I feel as though there is [a] scientific rationale... I think that the two rare instances of HIV cure occurring in patients who have gotten stem cell transplants provide some basis for believing that this is a possibility. I do think that there's hope, and this is potential innovation that could possibly lead to HIV “functional cure.”*—Regulator (#08)

*It would require a lot of discussion… ahead of time. And our technologies are advancing, things change a lot, quickly.*—Regulator (#06)

Three biomedical researchers (#05, #09, #19) commented that combination cell and gene modification products represent a promising approach towards HIV cure given recent scientific advancements. Cell and gene modification approaches that could be scalable to LMICs are also being explored (Biomedical Researcher, #19).

Nevertheless, all informants agreed that cell and gene modification necessitates enhanced safeguards. These safeguards included ensuring specificity of the products, testing for off-target effects, staggering interventions and trial participants, and having clear stopping rules in case of intolerable toxicity or futility. Several participants described the mandate for long-term monitoring of trial participants to assess potential for carcinogenicity, teratogenicity, and mutagenicity of cell and gene products.

#### Combining latency-reversing agents and immune-based interventions

For the most part, informants recognized the scientific rationale for combining LRAs with immune-based agents and further stated it was critical to combine them. However, some LRAs are repurposed compounds and associated with significant toxicities (Regulator, #08).

Some informants cautioned combining LRAs and immune-based interventions due to their potential for drug-drug interactions and unexpected safety outcomes.

*Let's think about this very carefully… You're perturbing a lot of systems there because latency reversal is not pinpointed… So, the off-target effects there can be quite big… That's where I would really say, "Stop and think very carefully." Not stop forever, but really just… make sure we know what we're doing.*—Policy Researcher (#04)

*The only thing, which again you come up to with all of these, is safety. If some of the latency reversing agents are messing around with DNA [deoxyribonucleic acid] methylation or other things, the same safety principles would apply regardless of whether it was for HIV, and that you're not inducing increased risk for hematologic malignancies, or what have you.*—Biomedical Researcher (#09)

Three biomedical researchers (#11, #13, #19) and two community members (#03, #05) noted LRAs have not proven effective at reducing the HIV reservoir to date. Two informants (Biomedical Researcher #05, Community Member #03) endorsed studying the “block and lock” approach, the opposite of latency reversal whereby the goal is to keep HIV dormant.

*At the moment, different LRAs have been tested and, to date, none had a large enough clinical effect to really lead to enough latency reversal that would be clinically relevant.*—Biomedical Researcher (#19)

*If it's dormant, why wake it up? And why wake it up and risk waking it up and not being able to make latent again? …*

*A lot of people think more about “block and lock.”*—Community Member (#03)

Several informants recommended performing case-by-case evaluations of LRA and immune-based intervention combinations, especially for repurposed cancer agents with known toxicities. Three biomedical researchers (#09, #11, #13) recommended implementing agent-specific safeguards.

*Well, so here there are two different sets of risks. The LRAs… may have their own specific potential toxicities. Some of them are known to be mutagenic, and so obviously that has some concerns and limits the kinds of populations in which they can be studied… So, safeguards involve starting with people who are basically healthy and having very close and careful monitoring… The second half of that, the immune-modulating agents, …the issues are the risks of those agents… I think there needs to be both careful consideration of appropriate inclusion and exclusion criteria based on the specific set of risks associated with the individual immune modifying agent and a careful monitoring and targeted monitoring, again, based on the potential risks.*—Biomedical Researcher (#02)

Additional LRA-associated safeguards included maintaining ART for LRA-only studies (Biomedical Researcher, #11) and long-term follow-up of trial participants (Community Member, #15).

### Additional considerations

We explored considerations related to: (1) ensuring improvement over standard ART, (2) challenges to combination HIV cure regimens, and (3) engaging communities around combination HIV cure research.

#### Ensuring improvement over standard ART for PLWH

We asked informants to describe what would be required in a combination HIV cure regimen to ensure a meaningful improvement over standard ART for PLWH. A regulator (#08) recommended defining the desired clinical benefits from a combination HIV cure regimen to clarify the selected strategy.

*You probably need to have a clinical trial demonstrating some sort of benefit. You also have to figure out what you want that benefit to be.*—Regulator (#08)

A biomedical researcher (#02) explained that a combination HIV cure regimen may not represent an improvement over existing ART safety profiles. Three biomedical researchers (#02, #07, #13) explained that PLWH would need to weigh the expected risks against any benefits from their individual perspectives.

*It's very unlikely that a cure will be an improvement on existing safety profiles of ART, which we know is extremely safe and people can take indefinitely. But there are improvements in that it removes the need to take treatment lifelong and therefore offers options for people… [Combination HIV cure strategies] may have a greater chance of a significant toxicity, but it will have the benefit of no ART lifelong. So, people will make those decisions based on the likelihood of getting a benefit. If the likelihood of the cure working is 20% or 80%, that will also determine how much risk people are going to accept.*—Biomedical Researcher (#07)

Several informants recommended integrating community perspectives to define what a meaningful clinical benefit would be. A biomedical researcher (#05) further advised considering issues of accessibility, affordability, and stigma for PLWH.

#### Challenges to combination HIV cure regimens

Informants discussed challenges to the design and implementation of combination HIV cure trials. A key limitation was getting pharmaceutical companies to collaborate in designing combination products. A biomedical researcher (#05) described the lack of incentives and recommended exploring public–private partnerships to spur innovation.

*Get[ting] companies to work together is really quite tricky, and that may be a bigger issue than the science… There's no incentive that I know of to help companies overcome that barrier… It may be that in [the] HIV cure space that that type of model that has come from the HIV vaccine field could be explored, how to get the few remaining companies… interested in HIV cure research, and say, "What can we do to incentivize the company through tax breaks or things that would allow for studies that would combine or compare?"*—Biomedical Researcher (#05)

Additional challenges included the lack of overall coordination in the field and the scarcity of shared outcomes across trials to allow researchers to make head-to-head comparisons.

*A more collaborative approach rather than competitive approach to these trials would probably be helpful… It would be helpful if there's consensus and some shared outcomes between trials so that you could compare them.*—Regulator (#08)

Biomedical researchers described trial design challenges such as long timelines, determining the optimal timing of the ATI, and the “dangers of doing these smaller uncontrolled experiments” (Biomedical Researcher, #02). A biomedical researcher (#05) recommended exploring small Phase 0 trials to shorten clinical development timelines.

*I don't see why they couldn't go into a small phase 0 trial in people under appropriate informed consent and safety conditions. I mean, otherwise you're just wasting another year or two doing your study in non-human primates… So not just an observational clinical study on patient samples, but a study designed in very small numbers, not to please the statisticians for statistically valid numbers, but just to test something for a bigger effect that's considered to be safe to give to people.—*Biomedical Researcher, #05

A biomedical researcher (#19) noted the need for a reliable assay that could predict loss of viral suppression when participants are off ART.

*Another big concern is that we don't have a great way to predict when someone would lose that control… Ideally there would be an assay, the same way that clinically we follow viral loads and CD4 counts over time…*—Biomedical Researcher #19

Biomedical researchers identified two additional hurdles: defining “clinical relevance” for PLWH (Biomedical Researcher, #09) and future commercialization of combination HIV cure products (Biomedical Researcher, #09).

#### Engaging communities around combination HIV cure research

Informants provided guidance on ways to meaningfully engage communities around combination HIV cure research. Priorities included clarifying expectations about HIV cure science and emphasizing the early-phase nature of current combination experiments. The need for research capacity building around the complexity of the science also emerged.

*You just have to be really honest about what the goals are; that this is far from product development, these are experimental clinical trials, experimental medicine. So you can't give people false hope. And being honest about the long-term unknown risks.*—Regulator (#06)

*Well, I think we need to do a better job of letting people know that we're doing this research and we're years away from a cure if ever, and really managing expectations and building research literacy.*—Community Member (#01)

Community members emphasized the need to build trust in the research and advised designing HIV cure trials with equity considerations in mind. There should be a plan in place for the equitable roll out of combination HIV cure regimens once these are available (Biomedical Researcher, #05).

*I've not seen a coordinated plan for discussion on roll out of an HIV cure when it is available. And you know, I'm a believer that there will be an HIV cure available… I can't think of any single WHO [World Health Organization] stamped document that describes how to have HIV cure roll out amongst populations from an equitable perspective.*—Biomedical Researcher #05

In sum, community and societal aspects of combination HIV cure research should also be carefully considered.

## Discussion

Developing combination HIV cure regimens for PLWH presents a distinct set of ethical, social, scientific, regulatory, and practical challenges. Informants in our study converged around combination HIV cure regimens likely being required to make meaningful scientific advancements towards an HIV cure [10]. They also generated ethical and practical considerations for designing and implementing combination HIV cure trials. This study extends the growing literature focused on advancing the understanding of critical ethical and social aspects of HIV cure research [25–29].

Combination HIV cure trials will remain critically important to advance the scientific search towards an HIV cure. At present, most combination HIV cure trials follow the ethics of experimental medicine [6, 30]. Because the prospect of direct clinical benefits in early-stage trials is small or non-existent, risks must be justified by the social value of the scientific knowledge to be generated [6, 31, 32]. Balancing and optimizing HIV cure combinations for safety and efficacy as part of trial designs is necessitated, while operating within the constraints of the clinical drug development process.

As noted by informants in our study, a critical challenge in the HIV cure field remains the lack of a clear biomarker to predict viral relapse [8]. Having well-defined clinical endpoints would move the field exponentially and provide a clearer licensure pathway towards an HIV cure. The efficiency and feasibility of HIV cure approaches will also be dependent upon thoughtful and expeditious ethical and regulatory reviews and approval. Although biomedical researchers identified potential gaps within the existing guidance for novel HIV cure interventions under development, they also acknowledged that the FDA is willing to consult with research teams on a case-by-case basis. Informants were satisfied with the FDA’s consultation process, collaborative regulatory reviews of combination HIV cure products across agencies, and receptivity towards combinatorial HIV cure science. A key challenge we have found in our practice, however, is conveying to potential participants the regulatory considerations that go into designing and implementing these complex combination HIV cure trials. For example, the intensive safety monitoring and regulatory oversight that characterize these studies may lead to frequent and/or unforeseen changes to the study schedule (e.g., need for extra visits to follow up minor abnormal results or study pauses related to safety events in other participants). Further work examining the experience of participants in these studies, particularly with regard to the complexity of safety monitoring and regulatory oversight and the optimal way to set expectations that unforeseen issues may affect their experience in the study, is warranted.

One important question remains around how combination HIV cure regimens could represent meaningful therapeutic improvements for PLWH over currently available ART. Informants noted that the expected clinical benefits of combinatorial regimens must be worth the associated risks and that regimens should fulfill unmet needs for PLWH. To ensure acceptability and tolerability of combination HIV cure regimens, we therefore call for robust empirical ethics and socio-behavioral research as part of ongoing combination HIV cure trials to better understand participant perspectives and tolerance for risks—both clinical and psychosocial—to ensure combination HIV cure regimens remain acceptable [33]. Our team is committed to understanding patient/participant perspectives around combination HIV cure trials.

A key gap identified by our study was the lack of meaningful opportunities to debate priority combination HIV cure regimens that should be tested in clinical trials. This finding has important implications for the field of HIV cure research given limited resources and potential eligible trial participants. More emphasis should be given towards generating consensus around priority combinations, and these should also be extensively vetted with community advisory groups.

Another important next step will be to continue refining and socializing target product profiles [34] for combination HIV cure regimens, with an eye towards real-world translation and implementation. Considerations will need to be paid to issues of scalability, accessibility, equity and affordability for PLWH. To maximize the important social value of an HIV cure, combination HIV cure regimens need to be translatable into evidence-based practice for clinical care for millions of PLWH worldwide [28, 34, 35].

Several challenges will doubtlessly need to be overcomed to create safe, effective and acceptable combination HIV cure regimens, such as ensuring collaboration among pharmaceutical companies, particularly around IP and reward issues [36]. In 1993, the Inter-Company Collaboration for AIDS Drug Development, a group of 15 pharmaceutical organizations, was created to propel the development of effective cART [37]. A similar initiative may be required to break down barriers towards designing effective combination HIV cure regimens.

### Limitations

Our findings should be interpreted in light of the following limitations. Funding and time constraints precluded interviewing additional informants, including bioethicists, HIV clinicians, and researchers working in similar fields, such as combination cancer research. Possible sampling bias was introduced because informants were self-selected following formal invitations to be interviewed. Most informants were supportive of combination HIV cure research, and dissenting views may have been underrepresented. There was a lack of diversity in our sample, particularly related to race and ethnicity of informants, since all were White/Caucasian. This is a major limitation of our study. A similar study will be needed with more diverse and non-US-based informants. After 19 interviews, it is possible that we did not reach saturation, the point when no new information emerges [38]. We did not inquire about ethical considerations related to interrupting HIV treatment in the context of combination HIV cure research, as these are reviewed extensively elsewhere [39–42]. Further, our research was not designed as a consensus study. Additional stakeholder engagement will be necessary to generate consensus around guidance for ethical combination HIV cure research. Despite these limitations, we presented the findings with fidelity to the data, and we believe our study to be internally valid.

## Conclusion

The increasing number of combination HIV cure trials brings with them a host of ethical and practical challenges. Our qualitative interview study yielded ethical and practical considerations for developing effective combination HIV cure regimens. With this paper, we hope to inform meaningful stakeholder dialogue around the use of combinatorial HIV cure research approaches. Triangulating perspectives of different stakeholders—including biomedical HIV cure researchers, community members, regulators, and bioethicists—will be critical to ensure HIV cure research remains ethical and acceptable. To protect the public trust in HIV cure research, considerations should be periodically revisited and updated with key stakeholder input as the science continues to rapidly advance (Additional file 1: Table S1).

## Supplementary Information

**Additional file 1: Table S1**. Additional Quotes—Ethical and Practical Considerations for Designing and Implementing Combination HIV Cure Trials (United States, 2020–2021).

## Data Availability

All relevant quotes have been included in the results section.

## Abbreviations

ART: Antiretroviral therapy
ATI: Analytical treatment interruption
AVRC: AntiViral Research Center
bNAbs: Broadly neutralizing antibodies
CAB: Community advisory board
CAPS: Center for AIDS Prevention Studies
cART: Combination antiretroviral therapy
CBER: Center for Biologics Evaluation and Research
CDER: Center for Drug Evaluation and Research
COVID-19: Coronavirus Disease 19
DARE: Delaney AIDS Research Enterprise
DNA: Deoxyribonucleic acid
FDA: Food and Drug Administration
HAART: Highly active antiretroviral therapy
HARP-PS: HIV + Aging Research Project-Palm Springs
HIPAA: Health Insurance Portability and Accountability Act
IND: Investigational New Drug
IP: Intellectual property
IRB: Institutional review board
LMIC: Low and middle income countries
LRA: Latency-reversing agent
PD: Pharmacodynamics
PK: Pharmacokinetics
PLWH: People living with HIV
UCSD: University of California San Diego
UCSF: University of California San Francisco
UNC-CH: University of North Carolina at Chapel Hill
US: United States
WHO: World Health Organization
