# Supplementary material for: Considerations for designing and implementing combination HIV cure trials: findings from a qualitative in-depth interview study in the United States
Source: AIDS Res Ther. 2021 Oct 18;18:75. doi: 10.1186/s12981-021-00401-8 (PMC8522863; doi:10.1186/s12981-021-00401-8)
Supplement: Supplementary file 1 — Additional file 1: Table S1. Additional Quotes—Ethical and Practical Considerations for Designing and Implementing Combination HIV Cure Trials (United States, 2020–2021). [file 12981_2021_401_MOESM1_ESM.docx]

**Table S1: Additional Quotes – Ethical and Practical Considerations for Designing and Implementing Combination HIV Cure Trials (United States, 2020 – 2021)**

| **Themes and Sub-Themes** | **Participant Number** | **Informant Type** | **Exemplar Quotes** |
| --- | --- | --- | --- |
| **Considerations for Combining Interventions Towards HIV Cure** | | | |
| **Ethical Considerations for Combination HIV Cure** | | | |
| Combinations will likely be required for durable ART-free control of HIV | 06 | Regulator | *I am also of the belief, like many, that combinations are going to be needed.* |
|  | 04 | Policy Researcher | *I think the reason we need to combine strategies will be because neither agent alone will actually achieve that.* |
|  | 05 | Biomedical Researcher | *But sometimes you might say, “Well how do you leap into the ocean when you haven't stepped in the pond?” and, you know, you just have to build a bigger ship and just get there quicker. So, I think that I'm in favor of combination antibody approaches, and the kitchen sink can become the kitchen sea.* |
|  | 18 | Biomedical Researcher | *I think it's very unlikely that we're going to come up with anything that is a single intervention with a single mechanism of action.* |
| Prioritization of combinations will increase social value | 14 | Bioethicist | *I'm not convinced we have very effective tools or techniques for selecting combination therapies for prioritization in research… A lot of the reasons why combination therapies often get tested have to do with relatively arbitrary variables, like whether a combination drug is on patent or whether a company is… interested in pursuing an extension or a label, [or] whether or not the IP is owned by [the same company]… I suspect there's a lot from a standpoint of social value… there's probably a lot of suboptimal decision-making that is partly a function of the fact that the scientific judgments are often embedded among a lot of economic and social judgments that have very limited direct bearing on the clinical question at hand.* |
|  | 02 | Biomedical Researcher | *There are so many different possible combinations that it's pretty clear we can't test them all in people. How you prioritize which combinations to test becomes a major challenge, particularly if you can't validate the combination in an animal model… Given this research is extraordinarily expensive and time consuming, and that you're asking an awful lot of the participants in the research as well, you really want to make sure that… you have some evidence… that this combination really makes sense to move into people.* |
| Strong scientific rationale for combining interventions | 09 | Biomedical Researcher | *I think you want to have things where there's a rationale… There are many, but one challenge, for example, is if you're combining an immune approach and a reservoir approach... when do you do those? Do you do those at the same time, do you do one before the other, how long do you wait? If there's an interval, do you have to do it more than once?* |
|  | 10 | Biomedical Researcher | *A lot of these strategies are paired specifically because we think that together they have a unique mechanism of action... That's the most straightforward sort of concept of how we combine things; … we think that they work together to do the ultimate task of identifying and clearing the reservoir.* |
|  | 11 | Biomedical Researcher | *I think the first thing that comes to mind is there has to be some plausible reason why these two things or three things will act synergistically.* |
|  | 12 | Biomedical Researcher | *All right, but the reality of the world is that you're going to have to have a strong scientific rationale because it's going to go to peer reviewers and funders and IRB and FDA, so that's the reality.* |
|  | 19 | Biomedical Researcher | *And see if the mechanism of action of the individual component of that combination strategy, if there has been sufficient supportive information from the lab, on how they would add to each other in terms of their activity.* |
|  | 03 | Community Member | *But all that should be in the rationale… I mean, I think we would alleviate a lot of the combination all-time problems if we could do them sequentially knowing what you think they would do and having a good rationale for that and knowing… in which order they should be administered.* |
|  | 15 | Community Member | *There has to be a sort of reasoning for why some things might combine together in a way that's going to lead to some kind of informative or beneficial outcome. You have to be sure that there's not going to be any negative interactions with the antiretroviral treatments that people are taking and, depending [on] who's going to be involved in the trial, making sure that all the toxicology is done to make sure it's safe to give to a broad population of people… I think at this stage, because it's so exploratory, you're not necessarily going to have a perfect sort of rationale necessarily for trying things, but perhaps it's a little bit inferential. It depends on the sort of circumstances to how you might justify it.* |
| Risk minimization and mitigation | 08 | Regulator | *Well, obviously, I already mentioned the potential for the two products or the two interventions to create added risks that might not be seen or maybe wouldn't be seen at as high enough frequency as it would be seen in a small study when you combine them. They could elevate those risks and multiply the risks.* |
|  | 02 | Biomedical Researcher | *Obviously, you want to be reasonably certain that combining approaches is not somehow going to dramatically escalate the risks of adverse events or toxicities.* |
|  | 09 | Biomedical Researcher | *I think you want to do the best due diligence you can to try to understand how do you mitigate that risk or how do you monitor that risk, both and either/or of doing that, because what you don't want to do is obviously end up again either setting the patient backwards in terms of their HIV reservoir size, if you will, or create untoward adverse effects in that way.* |
|  | 11 | Biomedical Researcher | *I think you'd be hard pressed to test a combination without having clinical experience with each of the agents individually first, and those two agents individually should both have good safety profiles.* |
|  | 12 | Biomedical Researcher | *It should be safe.* |
|  | 19 | Biomedical Researcher | *I think we would want to have an idea of the safety of the various modalities, at least by themselves.* |
|  | 01 | Community Member | *Obviously, they hope there's a synergy in terms of efficacy, but there also could be an adverse events as well.* |
|  | 17 | Community Member | *What are the side effects? How tolerable is tolerable? For what you would get in return?* |
| Characterization of drug-drug interactions | 04 | Policy Researcher | *If the strategies involve oral agents like a pill of one kind and a pill of another kind, or it can even be an injectable or whatever, but two different kinds of drugs, you obviously need to go do all of the drug-drug interaction… You can combine drug A with drug B and then do your little squares and concentrations and look at the different types of drug toxicity and drug-drug interactions. So that again is, and especially in immune modulators, the immune system is very complicated.* |
|  | 09 | Biomedical Researcher | *So, some of the standard ones that would apply, regardless of the mechanism, would be for example a small molecule agent, you'd have to understand potential drug-drug interaction profile with the HIV medicines that the individual's taking.* |
|  | 11 | Biomedical Researcher | *And then you'd also want to have done drug/drug interaction studies in appropriate preclinical models, again probably non-human primates… We definitely don't want to skip over a step of looking at that in preclinical safety studies, even if the two drugs individually have a fantastic safety record in the clinic.* |
|  | 03 | Community Member | *You have to know if there's drug-drug interactions.* |
| Robust informed consent about potential risks, including unknown risks | 14 | Bioethicist | *There are issues about when you combine drugs; generally, you get higher levels of safety issues. So, there are issues about managing safety on those trials and making sure that patients understand that, typically, you're going to get a higher safety signal on a combination therapy drug trial than you will on a mono trial.* |
|  | 08 | Regulator | *Then, of course, as we already discussed, the consent really needs to be well written to communicate the unknown and the reality and the likelihood that this intervention might not have this kind of cure, or functional cure, or any kind of benefit at all to that individual participant so that those participants are fully informed about the risks.* |
|  | 06 | Biomedical Researcher | *Again, this is really experimental medicine… It just really has to be explained well to volunteers that we're a long way from drug development.* |
|  | 10 | Biomedical Researcher | *The informed consent process just has to be incredibly robust and detailed, but clear and efficient discussions of what the actual risk is for a participant of that study.* |
|  | 18 | Biomedical Researcher | *And coupled with that is really rigorous informed consent. They're critical. And that doesn't mean more pages. It means assessment of whether people are informed, as best as you can.* |
| **Trial Design Considerations for Combination HIV Cure** | | | |
| Trade-off (or balance) between novel paradigms versus older approaches | 14 | Bioethicist | *I think that that trade-off between novel paradigm breaking approaches versus kind of tweaking and trying to get every last little drop out of already existing drugs is a big sort of social problem that we struggle with in drug development.* |
| Great potential for synergistic efficacy while minimizing safety concerns | 04 | Policy Researcher | *We can also have two approaches that could have [a] huge synergy impact on safety and really increase the safety concerns.* |
|  | 13 | Biomedical Researcher | *It shouldn't be just random combinations; it absolutely makes sense… to start with something that appears to make sense and seems to have the promise of a synergistic effect… I think it's very hard to anticipate and that's exactly why we need these clinical studies because there's no other way to really evaluate a therapy fully.* |
| Maximize safety and efficacy information from the trial | 08 | Regulator | *Having a trial design that allows [for] the collection of information that informs the safety and efficacy of each of those interventions. There are ways to design a study that might be more informative than others. For example, having some participants receive one intervention, some another, and then having other participants get the combination. That might be the kind of approach that would be more informative for both safety and efficacy.* |
| Careful inclusion/exclusion criteria | 09 | Biomedical Researcher | *Who are the individuals that could be allowed to participate in the study? I think that's really important, and including those, casting a wider net, if you will, or not being so restricted.* |
| Minimizing participant burdens | 14 | Bioethicist | *And you want to make sure that you are testing something that really, again, has good science behind it… [and] has [the] prospect of advancing further into clinical development. And it's not going to expose patients to undue burden and hardship for volunteering their bodies.* |
|  | 15 | Community Member | *I think you have to think about the participants and what is going to be involved for them. I don't think there's any researcher that wouldn't do that, but the practicalities can be important. And so visit schedules and all those kinds of issues related to what the logistical sort of things that you're asking people to do.* |
| Long-term follow-up of trial participants | 05 | Biomedical Researcher | *From a scientific perspective, combinations involve potentially changing safety profiles from one agent to adding in another, and then also the possibility for longer term impacts on studies which require continued surveillance and monitoring from a safety perspective and a potency perspective.* |
|  | 03 | Community Member | *It may be that you might want to do longer follow-up in combination trials… because most of these combination trials are about pumping up the immune system. And really, you want to see not only if that works in the long-term but for how long it might work, so that would require long-term follow-up.* |
| **Ensuring Acceptable Benefit/Risk Profiles** | | | |
| Robust pre-clinical evidence | 14 | Bioethicist | *So, have they done sufficient work in the preclinical setting to pick out this handful of combination of drugs with the right sort of doses, administration schedule, et cetera, to move into a phase I study.* |
|  | 06 | Regulator | *I do think there has to be some preclinical evidence to support each component… So, I think there's a lot of upfront preclinical stuff that you would look at individually before the combinations… But that every effort has been made to use what preclinical data we have to support going forward in first in humans.* |
|  | 08 | Regulator | *Whereas a non-human primate model, though expensive, really will be able to give you a better idea of what's going to happen and what's more likely to happen in humans [than humanized mice].* |
|  | 09 | Biomedical Researcher | *I think the only way to try to do that [maintain benefit/risk balance] is just through an appropriate pre-clinical safety and toxicology program… If you were bringing forward a novel entity that's never even been in humans, you'd probably have a little higher bar.* |
|  | 10 | Biomedical Researcher | *I think we want to test and move into human clinical trials studies that have a reasonable safety profile and some evidence of effect in a robust animal model.* |
|  | 11 | Biomedical Researcher | *So, I think there's a lot we can do in vitro and in preclinical models to explore those things kind of more thoroughly before going ahead with these clinical studies.* |
|  | 19 | Biomedical Researcher | *As far as activity, I think it is desirable to have at least pre-clinical in animal models or, depending on what the modality is, if it can't be tested in animals, in vitro that supports the idea that the combination is in fact adds value to having either component alone.* |
|  | 15 | Community Member | *I think they can help because there's an additional layer of complexity and potential risk associated with combinations. Then maybe there's a great need there for some kind of animal study to make sure that there's a reason for taking those risks.* |
| Ensuring safety of individual interventions | 19 | Biomedical Researcher | *I think safety is what really has to be, safety of the individual components and when they are in combinations, really where it has to be in the forefront of everything that we do.* |
| Maximizing scientific benefits while minimizing risks to trial participants | 18 | Biomedical Researcher | *Well, I think the question is, how do we minimize risk? Again, because the whole... I mean I guess there is the scientific benefit; that's the benefit that comes out of it… We're going to have really clear guidance on how we define benefit. And again, this is scientific benefit, not individual benefit.* |
| Reducing risk thresholds that can be tolerated in otherwise healthy PLWH | 09 | Biomedical Researcher | *This is, for the most part, people who, if they have access to therapy and they take therapy, can be doing quite well.* |
| Psychosocial benefits are important but difficult to measure | 12 | Biomedical Researcher | *If you think that the benefit you're getting for participating is that you're being altruistic, then how do you measure that? To now calculate the numerator, where that would balance out?* |
| **Regulatory Considerations for Combination HIV Cure** | | | |
| Regulation requires judgment | 14 | Bioethicist | *Essentially, FDA [or] some entity needs to make a judgment about the social value against the risk, and assessing social value requires judging the quality and maturity of the science behind a phase I study, which is a very technical judgment… The quality of the design, whether or not the trial design is such that if this intervention has quality, it's going to detect it.* |
| Individual participant determination of social value  (i.e., Voice of the Patient) | 04 | Policy Researcher | *Regulation is all about benefit and risk, and not everyone sees it the same way… You have to make a judgment at some point that this benefit is enough and this risk is not too much. If you're a patient, you might think about it very differently than if you're a sponsor who's going to sell the drug or a regulator who has to make that decision. On top of that benefit and risk, we have this uncertainty, and it's always how much uncertainty about what might happen are we really willing to live with. That's, again, a question where the community, the patient voice really needs to be heard because some patients might be happy with a lot of uncertainty and others want to know very clear.* |
|  | 04 | Policy Researcher | *If I go through this, I will be 100% cured. There's not a chance that I will not be cured, and there needs to 100% chance that I won't have any damage." Other patients might say, "Well, if I have a 50% chance of being cured but maybe I'll have some scarring of the liver or some kind of a long-term consequence, that's okay with me.* |
| Existing FDA guidance on designing combination trials and FDA pre-IND process | 06 | Regulator | *It would be good as always to have early pre-IND meetings with the agency to go through what pre-clinicals would be needed in the toxicology studies and things like that. So again, we need to get down to what the specific agents are and the target population and all that. The earlier you can interface with the agency, we can provide our guidance and the way we're thinking about it, and you can educate us about what and why you're going in this direction, so that there's a common understanding of what's trying to be done and what we know about the agents.* |
|  | 04 | Policy Researcher | *Again, the FDA for example has guidance on an antiviral that's being used in combination. So, that all is very clear and I think there, again, it is dependent a little bit on what you're combining with what.* |
|  | 13 | Biomedical Researcher | *I would assume there is a risk that you have more side effects than just the sum of the side effects of the two interventions together. So I think there should be a higher bar and IRBs and regulatory agencies should look much closer on these [combination] therapies.* |
|  | 03 | Community Member | *There are different guidance about combinations therapy.* |
| Ensuring robust safety data in humans for individual therapies before moving to combinations | 12 | Biomedical Researcher | *I guess, if something has never been in humans before, you would not leap straight into a combination.* |
|  | 19 | Biomedical Researcher | *So, having human data with the antibodies alone and human data with this other molecule alone was sufficient for the FDA to bring the two together.* |
| Ensuring intended proximal biological effect | 06 | Regulator | *You want to know that each component is contributing to the effect, otherwise you only have risks.* |
|  | 02 | Biomedical Researcher | *I think that becomes a regulatory issue, that you need some reasonable evidence that each agent has at least its intended proximal biological effect. And then hope when you combine them that they're having the effect on the HIV reservoir that you're looking for.* |
| Precautionary principle when evaluating combinations | 14 | Bioethicist | *So that one rogue investigator or some sort of ill-conceived set of clinical trials doesn't undermine confidence such that it sets back other much more legitimate efforts in a research area.* |
| **Determining Individual and Combination Effects of Interventions** | | | |
| Critical to know the effect of individual components | 08 | Regulator | *Ultimately, from a regulatory standpoint, it's very important to show the contributions of the individual agent…Therefore, you would really need to justify that that product or intervention itself is really integral to the effects that you're observing.* |
|  | 14 | Bioethicist | *I think you generally would want to be able to show activity for each of the drugs by itself.* |
|  | 02 | Biomedical Researcher | *The other issue is about activity: that you need some evidence that each intervention has independently the intended effect because, if you don't know enough about the agents and you just put them together and then you say, "See, this combination worked," how do you know that the one alone wouldn't have worked just as well?* |
|  | 19 | Biomedical Researcher | *Having safety data on the individual components, even if it's not in the context of HIV, …is something that it's important to have because… we are going [in]to a healthy population with these molecules that have direct effects in their immune responses.* |
|  | 19 | Biomedical Researcher | *I think it's important to know... it goes back to how large our studies need to be, right?* |
|  | 01 | Community Member | *Absolutely. Well, you need to know a, what the risks are, and b, how well it's going to work one by one before you start combining them.* |
|  | 03 | Community Member | *You should absolutely know that sort of stuff about these cure interventions or you're shooting in the dark.* |
| Having additive or synergistic effects is ideal | 04 | Policy Researcher | *So, what signal are we looking for to say that combining A and B would actually produce more than either A or B isolated?* |
|  | 11 | Biomedical Researcher | *I think there's a lot that can be done there and a lot that the field has learned over the years. So as one example, a lot of the “kick” agents have either synergistic or antagonistic effects with the kill agent with cytotoxic T-cells.* |
| Difference between additive versus synergistic effects is less critical | 12 | Biomedical Researcher | *So between synergism and additivity, no, I don't.* |
|  | 19 | Biomedical Researcher | *If it has to be synergistic versus additive, I don't know. I think additive is good enough for where we are at this moment.* |
| Exclusion for antagonism | 02 | Biomedical Researcher | *I think it would be important to exclude, to the extent possible, antagonism because that would be potentially… harmful to the individual. It would at least be pointless to combine approaches that could cancel each other out... I can't think a priori of cure approaches that would be likely to be antagonistic.* |
|  | 02 | Biomedical Researcher | *In the therapeutic realm, that was a concern about certain drugs, where they might compete for the same activation pathways and therefore shouldn't be combined.* |
|  | 09 | Biomedical Researcher | *I think where you would definitely want to have some information is if you felt there was some expected antagonism… But I think if you expect antagonism, you should either not go there or verify that, yes indeed, this is antagonistic.* |
|  | 12 | Biomedical Researcher | *So I mean, if they were antagonistic, unless that was actually your desired mechanism of action, that would be a problem.* |
| **Prioritizing Combinations to be Tested in Humans** | | | |
| Resources are limited | 10 | Biomedical Researcher | *I think the other issue is that we would like to test all the things in all the combinations, but there's just limits on the amount of clinical trial resources and funding and bandwidth that the cure groups really have, and so we need to prioritize. Especially since many of these trials are years long, it takes multiple years to screen and give these long-acting or staged strategies and then to wait for them all to go away or wait for these immune responses to rise before we do a treatment interruption, which in and of itself lasts another however many months.* |
|  | 15 | Community Member | *Unfortunately, resources aren't unlimited. And so I think you have to focus on where there's some sort of biological evidence that it's going to contribute to the outcome you want, whether it's reducing the reservoir or boosting the ability of the immune system's control or viral replication or both.* |
| Prioritize combinations with the highest scientific rationale | 08 | Regulator | *I would probably go with the agents that have the strongest scientific rationale…, which scientific approaches are going to be more likely to result in functional cure or other benefits.* |
|  | 04 | Policy Researcher | *The ones that have the most scientific validity behind them.* |
|  | 13 | Biomedical Researcher | *I think it's… driven by ideas of individual researchers and agencies and also of course the pharmaceutical industry… I don't see any forum where everybody comes together and gives their input.* |
|  | 19 | Biomedical Researcher | *I would say that, as a field, that [it] would be to the interest of all of us that we avoid overlap of tests in the same strategy, in the same clinical scenarios… We have to be very mindful of how we access those populations for the studies that we are going to be doing.* |
| **Determining the Number of Interventions to be Included in Combination HIV Cure Regimens** | | | |
| Acceptable number of interventions depends on the combination | 08 | Regulator | *I'm not sure that there is any acceptable number of strategies. However, it goes back to what's needed to really establish the safety and efficacy of each of these agents.* |
|  | 04 | Policy Researcher | *I mean without knowing what the strategies [are, it] would be, it's very hard to say.* |
|  | 12 | Biomedical Researcher | *It depends. I don't think I have an upper limit.* |
|  | 19 | Biomedical Researcher | *I don't think that there is a magic number of how many interventions should be combined. I think as a whole we have to look at these protocols and then try to simplify where we think we can, so that it can actually be done.* |
| Proceed in a stepwise fashion | 11 | Biomedical Researcher | *But I tend to prefer to approach it in a more slow and steady way and build into two and three agent combinations… I guess I was just saying my perspective is I think there's a lot that can be done with two and three agent combinations before we need to throw the kitchen sink, but everyone has different perspectives I guess.* |
| Skepticism beyond 3 interventions or modalities used in combination | 18 | Biomedical Researcher | *If you go more than three agents or four agents, you're not really going to be able to dissect out the contribution of the various components.* |
| No upper limit on number of interventions | 05 | Biomedical Researcher | *I would say as many strategies as will get to an HIV cure.* |
|  | 10 | Biomedical Researcher | *I don't think there's a finite cap. I don't think we need to put a number on that, but they obviously become more and more complicated to interpret specific effects. So hopefully in this iterative process, we may get to a point where we have this three-agent strategy does this and we've seen it consistently. And then we hope to build on that by adding this agent and then maybe the next agent.... So I don't think there is a necessarily a finite cap, but I also think it makes sense to be able to really understand what each of those interventions does from a safety and activity profile.* |
|  | 13 | Biomedical Researcher | *I don't think there is an upper limit. I mean, the question would be, “What do you define as a strategy?”* |
| **Considerations for Sequencing or Timing of Interventions** | | | |
| Contingent on interventions being tested | 06 | Regulator | *Well, it may depend.* |
|  | 04 | Policy Researcher | *That will be completely dependent on their mechanism of action and the rationale.* |
|  | 02 | Biomedical Researcher | *Well, I think it certainly depends, but that is a key issue. And, it depends because it's really going to be based on the intervention.* |
|  | 09 | Biomedical Researcher | *It may come down to what is it that you're doing, and how is the therapy administered, in terms of that… But it may vary with what it is you're trying to do, because like we said earlier, mechanistically, it might not make sense.* |
|  | 10 | Biomedical Researcher | *I think that totally depends on the mechanism of action or the proposed activity.... So depending on the mechanism of action, I think it's very rational or reasonable to design either all at once, everything together, or these sort of step-wise approaches. That just depends… But it would be nice from a safety perspective to be able to separate their administration so that you could have a sense of any sort of adverse events and what they are more likely related to.* |
|  | 11 | Biomedical Researcher | *But I suppose the other consideration depending on the agents involved, or what gives the lowest risk, right? So if the one agent, if you're kind of more concerned about that from a side effect profile, probably I guess you would give that first before deciding whether or not to come in with the second agent.* |
|  | 12 | Biomedical Researcher | *Totally depends what the interventions are… It would be based on biological mechanism of action, but the reality is that you also have to take into account tolerability.* |
|  | 13 | Biomedical Researcher | *That is something that has to be looked at very individually… You have to look at everything individually and make a decision based on the science and based on potential side effect profile, how to combine them, and if it might make sense to study them sequentially.* |
|  | 19 | Biomedical Researcher | *I think it depends on what's the effect that you want to have, right?* |
|  | 15 | Community Member | *There's definitely not going to be a one size fits all there, I think. It's going to depend on sort of what you're hoping to get… So there's issues around the sort of biology of each intervention that are going to partly dictate whether you can go all in or have to space out. And there's not going to be many things that are completely free of side effects. And you're not going to want somebody to have side effects of all interventions at the same time probably.* |
| Sequential administration advantages and examples | 06 | Regulator | *There is an advantage of doing them sequentially and some people may think that's required… The advantage would be that you can get your safety profile on that agent and you can track the immune marker… So certainly like a vaccine, for example, might be worth doing individually. You've got a way to track the immune response you want, to know that you've optimized that dose or schedule or whatever, and you don't have all this interference and confounding information coming from your other components.* |
|  | 08 | Regulator | *Well, from a safety and efficacy standpoint, it's better to give it in sequence.* |
|  | 11 | Biomedical Researcher | *You give yourself not only more chance to stop giving additional interventions and exacerbating reactions that are happening, and also to identify what's actually causing those issues if you stagger things. So, I think it's probably pretty standard to stagger these to some degree, and it's pretty hard to justify the other way around I think.* |
|  | 19 | Biomedical Researcher | *If you want to do vaccine and antibodies..., because the antibody can enhance the immune responses, so you would do something in sequence where the vaccine would modulate the immune responses in some fashion and then the antibodies come and… do their modulation also in a different way.* |
|  | 15 | Community Member | *So therapeutic vaccines are always going to need to be spaced out just because of the sort of the base or the way that they induce immune responses. And you don't want to vaccinate too frequently; you can actually kind of exhaust the immune response.* |
| Interventions need to be translatable and implementable in the real-world | 08 | Regulator | *I mean, that's certainly true with a combined antiretroviral therapy. Giving an intervention one after the other is actually not going to benefit anyone in the end. We've learned that over the years.* |
|  | 09 | Biomedical Researcher | *Ideally, it'd be better to have it all done at once, right? … I think ideally you'd want to minimize the burden to the person receiving it.* |
|  | 01 | Community Member | *And obviously you want to make things as user-friendly for the participants because, after a while, you're not going to be able to enroll trials of any size [to] actually prove if it works if it's too onerous for the participants. So that's all part of the equation, right? You have to weigh all these things out, but if something is going to be impossible to do in the real world, I think they really need to rethink whether that's approach they take or at least start thinking ahead of how can we modify this to make it more practical.* |
| **Considerations for Sequencing or Timing Interventions** | | | |
| Timing of interventions depends on the mechanism of action | 04 | Policy Researcher | *Well again, I think it's a lot dependent on the mechanism of action. If your step A needs to reach a certain point before your step B can act, then obviously we need to know how long that should be before you do the second one.* |
|  | 10 | Biomedical Researcher | *It does depend on the proposed mechanism of action.* |
|  | 11 | Biomedical Researcher | *The way that I would first think about it is, again, just a rational approach based on the known mechanism of action…, otherwise you would miss the window of opportunity.* |
| Need to rely on robust PK/PD data | 08 | Regulator | *It has to be from the science... For example, for a small molecule, it would be the PK but also animal study data… informing the design of the sequencing.* |
|  | 02 | Biomedical Researcher | *But I think it will depend entirely on which combinations, and that's a whole other set of potential experiments that may need to be done to optimize the timing or sequencing of the multiple interventions because, just as with the vaccine, you need to figure out what's the right dosing interval and how many boosters do you need.* |
|  | 12 | Biomedical Researcher | *So you might have a vaccine that you think that the peak of it inducing T cell responses or antibodies or whatever it is that you want your vaccine to do, that the peak of that is going to happen one week after you administer the vaccine and that's exactly the point at which you want to hit the immune system with… And you're just trying to find the optimal point at which, whatever the response is, is good enough that it's going to combine with the other thing that it depends on PK/PD.* |
| Timing around hours or days for LRAs | 09 | Biomedical Researcher | *If you look at the combination of the latency reversing agent and an immune mediated approach, you would think you would want to turn on the virus first and then have the immune activity occurring… But I think understanding more about the dynamics of these things in humans, just what's happening with the reservoir, what are normal cycles of ... they used to call it blips for plasma viral load, but for blips and virus expression at the reservoir level?* |
| Need robust modeling methods to determine optimal timing of interventions | 09 | Biomedical  Researcher | *I think there's just more biology to understand of the infection, but I think you want to, as best you can, upfront model what you think might make the most sense from studies in animals, and then you design into your first-in-human, your first in HIV positive human analyses to at least start to get a glimpse. There's going to be a lot of noise because there's a biological noise, et cetera, but you're just trying to get a feel for timing and kinetics before you go and do the same thing in hundreds of people.* |
| Oncology field can provide a precedent and guidance | 13 | Biomedical Researcher | *I think again cancer therapy might guide us a little bit in the general ideas of that.* |
| Optimizing timing of interventions | 02 | Biomedical Researcher | *For most things, I think we're looking at days, weeks, and months in terms of timing. I don't think there's a split-second timing that hours are going to matter here for the most part. It may be, for example, with some of the LRAs or even with some of the checkpoint inhibitors, that monitoring response needs to be done within hours or days in order to know that you've had the pharmacodynamic response that you hope for.* |
|  | 15 | Community Member | *So with a vaccine, you're going to know that it's going to take a month or so for the response that you've triggered to actually sort of do, I think, what's called differentiation, where you sort of trigger the right T cell to respond to what you want it to respond to, but then it has to go through a process of proliferation and its genetic machinery changes to start making all the sort of cytokines and stuff that you need.* |
| **Considerations for Specific Combination HIV Cure Regimens** | | | |
| **Combining Broadly Neutralizing Antibodies** | | | |
| bNAbs can be used in combinations due to safety | 08 | Regulator | *The safety profile of broadly neutralizing antibodies is favorable in comparison to a lot of other agents which have been repurposed in large part… repurposed products used for the treatment of malignancy or transplant.* |
|  | 04 | Policy Researcher | *I would think broadly neutralizing monoclonal antibodies are reasonably safe.* |
|  | 02 | Biomedical Researcher | *I think combination bNAbs are among the easiest to put together because there's no reason a priori to be concerned about their safety.* |
|  | 05 | Biomedical Researcher | *So in terms of combination antibodies, to me it's probably less of a problem… I haven't really seen any huge red flags from wanting to try that in terms of having different monoclonal antibodies together.* |
|  | 11 | Biomedical Researcher | *[bNAbs have] such a good safety record, such plausible modes of synergistic action with other agents.* |
|  | 01 | Community Member | *[Broadly neutralizing antibodies have] got a pretty good safety profile and they've been shown to be very effective in other disease areas and to have strong signals in HIV.* |
|  | 15 | Community Member | *They were seen [as] very safe and they're derived from people's own B cells originally. That seems to be a strong rationale for including them.* |
| bNAbs are viable components of future combination HIV cure strategy | 07 | Biomedical Researcher | *I think combination broadly neutralizing antibodies are unlikely to do anything on their own, but they are certainly a viable component of a future strategy.* |
|  | 10 | Biomedical Researcher | *The thing about bNAbs is they are well-characterized, both in HIV and in other sorts of diseases. They are human derived [and] they're well-characterized in both pre-clinical and phase I studies. And so for the most part, I think that bNAbs are easy things to add in combination because they are believed to be quite safe and have a specific function that is defined and then possible other functions that have yet to be fully described... They're being used almost exclusively for cure strategies in combination in order to be sort of like a backbone of a combination strategy.* |
|  | 13 | Biomedical Researcher | *So I think they are extremely promising.* |
|  | 19 | Biomedical Researcher | *I think it is fair to say that probably bNAbs will, from a cure point of view, it will be more likely for them to be successful if they are combined with other strategies. And then the other strategies can be vaccination, can be cytokine modulators in different forms.* |
| High costs of bNAbs | 09 | Biomedical Researcher | *There's been pushback on that because of cost and other things.* |
| bNAb safeguards to prevent resistance | 02 | Biomedical Researcher | *The main safeguard I think is ensuring that you don't generate resistance to the bNAbs at the level of the virus. That is a concern principally if these same bNAbs were to move forwards as therapeutics, say, for example, long acting treatment. And is there a risk that participants who are exposed to these bNAbs in cure trials develop resistance, say, during a treatment interruption, and then don't have this as a potential option down the road for as long acting.* |
|  | 05 | Biomedical Researcher | *I think that the concerns of a viral escape from antibodies is real. Combinations again reduce that, and the more combinations the better.* |
|  | 09 | Biomedical Researcher | *But I think you'd want to at least have something that has broad recognition and has some evidence of a little more challenge for the virus to develop resistance in vitro in that way.* |
|  | 11 | Biomedical Researcher | *I'm a fan of prescreening to make sure that people actually have virus that's susceptible to the bNAb that you're testing… I mean combinations with other bNAbs is probably the best safeguard against that, and not only in combinations, but in combinations where care is taken to make sure that you don't have a scenario where bNAb one washes out months before bNAb two and then you essentially have monotherapy.* |
|  | 19 | Biomedical Researcher | *Using combinations instead of a single antibody will protect from selection of resistance… But when you're thinking about bNAbs to be given in the absence of ART, you should have at least two [and] if possible three… That's why there is a value in the tri-specific antibody because one of the goals is to prevent selection of resistance… If you will transition into an ATI period to adjust the dose of those antibodies so that the decay of the combined antibodies is similar, so you're not in a situation where you end up with an antibody monotherapy. And the other point is shortening the ATI... [and] the viremic period, because before one goes back on ART, will that minimize the risk of selecting resistance.* |
| Discuss potential for bNAb resistance in the informed consent process | 10 | Biomedical Researcher | *Yeah, so I think that the theoretical risk that a participant could develop resistance to a class of antibodies that then would ultimately be an integral part of a cure strategy, I think that's something that should be included in an informed consent process.* |
|  | 15 | Community Member | *But I do remember that there was something about the resistance issue and I think that is important where people are getting a single or double antibodies, that they're at least warned that it might lead to them getting resistance and they wouldn't be able to use that antibody in the future if it became therapeutic or something like that.* |
| Additional bNAb safeguards | 04 | Policy Researcher | Monitoring for allergic reactions*: Of course, we know that people do have anaphylactic reactions and you can produce antibodies to the antibodies. Some of those risks are very well known and can be really looked for and monitored… Except if there's some kind of an interaction between the antibodies and the HIV that somehow leads to some kind of an antibody antigen interaction that somehow does something to other parts of the immune system. I guess continuous monitoring of the viral population.* |
| **Combining Cell and Gene Modification Products** | | | |
| Caution when combining cell and gene modification approaches | 06 | Regulator | *I think the field should be open-minded, but cautious.* |
|  | 05 | Biomedical Researcher | *You have to be more cautious.* |
|  | 11 | Biomedical Researcher | *But I would take a more cautious approach to gene therapy in the setting of otherwise healthy people on long term antiretroviral therapy.* |
|  | 13 | Biomedical Researcher | *I think caution is the most important thing here because these are totally new strategies.* |
|  | 01 | Community Member | *Yes, but very cautiously... We've seen it before and [it] could be very devastating.* |
| Cell and gene modification represents a promising approach towards HIV cure | 05 | Biomedical Researcher | *I think they're very powerful candidates because we understand that the people who have been cured of HIV are those that have basically shut off the receptor… Gene editing I think is a powerful approach… I don't think it should stop the development of a promising approach… I am a believer in gene editing approaches. I'm also a believer in gene editing approaches that combine with other approaches.* |
|  | 09 | Biomedical Researcher | *The advances made for cell and gene therapy in the last 10 years, even five years, there's been a lot of advancement where people are beginning to further optimize how and what's done.* |
|  | 19 | Biomedical Researcher | *It could be conceivable of launching in lower income countries… I think there is promise. If gene editing, it depends on the gene editing that you're talking about.* |
| Considerations for types of cells being modified | 02 | Biomedical Researcher | *So the issue becomes, are you working with mature T cells or with stem cells? The stem cell work is thought to have a greater potential risk.* |
| Depends on cell and gene modification approaches | 11 | Biomedical Researcher | *Certainly, anything with CRISPR gene modification I would not even contemplate doing that in combination at this point. You know, let's do that one at a time.* |
|  | 12 | Biomedical Researcher | *Then it comes down to what you're combining, right? … So I think it's more about how you deliver it.* |
|  | 13 | Biomedical Researcher | *I think it makes a difference also what kind of approach you use. Are you preparing cells, manipulating them ex vivo, and then re infusing them? And I think you have more control oftentimes with a strategy like this.* |
| Cell and gene modification safeguards | 19 | Biomedical Researcher | Enhanced safeguards: *That is because it's gene editing the safety guards I think have to be even more enhanced, because we don't really truly understand the long-term effect that this could have.* |
|  | 02 | Biomedical Researcher | FDA and DSMB oversight: *There is oversight from the… FDA… And I think key to all of these approaches is that these studies, even when they're small, pilot studies, really should have an independent data safety monitoring committee that can look at the data and provide an independent assessment about whether it's appropriate for the study to continue should a serious adverse event occur.* |
|  | 13 | Biomedical Researcher | Involvement of ethics committees: *It's very important to involve ethics committees.* |
|  | 11 | Biomedical Researcher | Stepwise approach: *I guess a progressive approach on that front seems like the best way to ensure long term success.* |
|  | 18 | Biomedical Researcher | Stepwise approach: *But I think they should be studied as single agents initially… But I think initially, they can be studied alone.* |
|  | 01 | Community Member | Stepwise approach: *So I think that's the one where you take a very cautious step-wise approach and try one and then the other, and then do them in animals and so forth before you even think about combining them in humans.* |
|  | 03 | Community Member | Stepwise approach*: I mean, they're dangerous enough individually. Let's not get the cart before the horse here. That's a big safety issue for me. That's a big red flag.* |
|  | 06 | Regulator | Monitor for off-target effects: *It's how well the off-target effects of gene editing are still being worked out and so, therefore, there are these long-term safety concerns.* |
|  | 09 | Regulator | Monitor for off-target effects: *The generic safeguards where you're looking at off-target either integration or immune activation, cytokine storms… all those kind of things.* |
|  | 12 | Biomedical Researcher | Monitor for off-target effects: *You have to work out that you are sufficiently on target and not off target. You have to look at teratogenicity.* |
|  | 08 | Regulator | Staggering interventions/participants and stopping rules: *There should be space between each, or time elapsed with monitoring between dosing, between individual participants. Obviously, the consents need to be very carefully worded. There needs to proper stopping rules.* |
|  | 15 | Community Member | Specificity of interventions*: So just having a clear sense of the specificity of the intervention and that it's not going to do any harm whether it could be used in combination might depend on how it's being delivered.* |
|  | 08 | Regulator | Enhanced informed consent: *There have been many genetically modified products that have not worked thus far. Therefore, I think it's very important to carefully communicate the potential and lack of high hopes that any individual approach will be successful even if it's possible. In addition, gene therapies, of course, pose added risks. Because unknown risks in a lot of ways that we don't know, a lot of the vectors, we don't know how long these cells are going to persist and we don't know what the impact may be in those trial participants. These are other concerns that should be communicated.* |
|  | 06 | Regulator | Long-term monitoring of participants: *We're really wanting to follow the gene therapy guideline of following people these 10 years or so… It still requires a huge commitment.* |
|  | 02 | Biomedical Researcher | Long-term monitoring of participants: *I think that that's where there needs to be careful, long-term follow-up of participants to ensure that there haven't been downstream consequences.* |
|  | 13 | Biomedical Researcher | Long-term monitoring of participants: *One of the things that people are worried about is potentially you could induce cancer, or you could induce other harmful consequences in the host, and I again think these studies have to be planned very carefully… And I think we need definitely long term data on these people.* |
| **Combining Latency-Reversing Agents and Immune-Based Interventions** | | | |
| Scientific rationale to combine LRAs and immune-based interventions | 02 | Biomedical Researcher | *So, one example is you could imagine some immune based mechanism for targeting reservoir cells that by itself isn't going to have any effect on the reservoir unless you stimulate the reservoir to be expressed or unless you first stimulate immunity with a vaccine and then enhance the function of the immune cells... It's only when you combine them, and that's where I think the proximate biological activity becomes the important marker as going forward, the criteria in that.* |
|  | 10 | Biomedical Researcher | *I do think that the concept of latency reversal plus an immune modulator is something that the field is excited about… So yeah, I think these are candidates to be put together. And it is important to think about whether it's through two agents or one agent, both the latency reversal activity as well as the immune modulation activity, and they both need to be characterized in isolation as well as together.* |
|  | 19 | Biomedical Researcher | *I think latency reversal would have to be combined with an immune modulator, because I'm a believer that the size of the reservoir may not be really our biggest problem, but really the composition of that reservoir. So if we are able to reset the immune responses in such a way that we can control those clones that continue to exist within the reservoir and maintain them unable to replicate, I think we may be fine. So, I don't think it's necessarily a number of reservoir cells but, rather, what's within the reservoir that is completely blocked in some way from replicating.* |
| Potential compunded toxicities of LRA combinations | 08 | Regulator | *There's scientific rationale… [for] latency reversal agents. A lot of them are repurposed drugs used for the treatment of cancer and are associated with a lot of adverse events or toxicities, and some of those toxicities are tolerable for patients or acceptable for a patient who has malignancy or a disseminated malignancy with low options in terms of other treatment options.* |
|  | 18 | Biomedical Researcher | *Based on what we know now, latency-reversing agents will ultimately have to be using combination, but they certainly can be studied as single agents to find out whether they… they do activate viral expression.* |
| Cautions around combinations involving LRAs (e.g., drug-drug interactions) | 07 | Biomedical Researcher | *So combination latency reversing agents may make sense biologically, but every drug has a capacity for drug-drug interactions and, so, that would need to be worked out first, in addition to having biological plausibility… So you've got to see whether that combination latency reversal is safe and actually is effective, and then you'd consider putting it with something else.* |
| LRAs have not proven effective at reducing HIV reservoir so far | 11 | Biomedical Researcher | *And our experience in doing that has been that we come in with strong LRAs and strong CTLs, and we measure the reservoir that's left, and it hasn't changed usually.* |
|  | 13 | Biomedical Researcher | *So far the strategies have been somewhat disappointing because I think the initial idea was that you reverse latency [of] all the cells that have HIV activated and, then, that you can help the immune system to kill all these infected cells. And we know now that it's not that straightforward. We can, with the current agents only, activate a very limited number of cells.* |
|  | 03 | Community Member | *It's not only that they haven't been able to find a decent LRA at this point…* |
|  | 05 | Community Member | *There's been a lot of studies, and the issue there has not really been safety, thankfully; it's just that they haven't really worked. So, I think it's an example where sometimes what happens in the laboratory isn't really necessarily predictive of what's going to happen in people but, at the same time, the people that have participated in those studies have done something important because that's the lesson that has to be learned.* |
| Support for “block and lock” approaches instead of latency reversal | 05 | Biomedical Researcher | *All the work done on the alternative approach, or one of the alternative approaches, of silencing the virus. I call it silencing into submission and then working out how you might combine its silence with then a wake up to see if there's any left, and should it be silenced, reversal silenced.* |
| LRA and immune-based intervention safeguards | 01 | Community Member | FDA oversight and community involvement: *I think you need to be extra cautious with those and… employ the stepwise approach and nonhuman primates and so forth to prove safety and efficacy before you would even think about going into humans… I think the FDA is really good about these, but I think what would help the field is to have community people at the table for those discussions… It's a conversation [that] needs to be had.* |
|  | 06 | Regulator | Case-by-case evaluation: *It's all case by case how it's designed with the preclinical is, what the follow-up is, what your target population is. And it's going to be a balance of that.* |
|  | 08 | Regulator | Case-by-case evaluation: *Therefore, those latency reversal agents might be acceptable to that patient population but might not be appropriate for patients who have well controlled HIV. Then, for immune modulatory agents, there's a lot of different kinds of immune modulatory agents. These have potential risks that might be different in HIV and patients living with HIV.* |
|  | 12 | Biomedical Researcher | Case-by-case evaluation: *They wouldn't be as high on my list, but it depends on the LRA and it depends on the immune modifying agent, right? So, if you had an LRA that worked better than any I've seen and if your immune agent was a CAR [Chimeric Antigen Receptor] T-Cell or a CAR-NK [Natural Killer] cell or CAR stem cell, I'd be more excited than if your immune agent was a therapeutic vaccine.* |
|  | 18 | Biomedical Researcher | Case-by-case evaluation: *I think an easy answer is yes, there should be. But I think it's really specific to what you're trying to put together, and there are some surprising results. I mean, the fact that when Gilead gave vesatolimod with the neutralizing antibody, they had some toxicity that was unexpected. Now, could have coincidental, might have been true, but not related. But that was a surprise because I don't think anybody thought, "Well, jeez... These two things.” One, either you have a lot of vesatolimod data from hepatitis B, and people thought, "Well, these antibodies are pretty safe in general." But I think it depends on the agents. The simple answer is yes. The detail is really hard.* |
|  | 08 | Regulator | Trial design considerations – staggering dosing and clear stopping rules: *In addition to stopping rules, providing that the dosing of patients, there should be time in between dosing that corresponds to the potential toxicities of those agents.* |
|  | 04 | Policy Researcher | Learning as much as possible about the risks: W*ith the latency reversal agents, whatever we know about them this far in terms of the potential toxicities and harms that they can do.* |
|  | 09 | Biomedical Researcher | Agent-specific safeguards: *Just have the safeguards of the inherent activity, so for example, the immune component: Is this something where they need to be at a hospital inpatient setting to take this therapy for a certain number of days because of a risk of something, cytokine whatever, or some other effect? ... I think it's that kind of thing and, again, it may be agent and/or mechanism specific.* |
|  | 11 | Biomedical Researcher | Agent-specific safeguards: *I guess the only thing that's really coming to mind is just being particularly sensitive to the potential issues that come with cytokine storm and general immune activation and trying to either disconnect that from latency reversal or being really cautious in terms of thinking of the threshold that's reasonable for an in vivo kick.* |
|  | 13 | Biomedical Researcher | Agent-specific safeguards: W*e also have the specific mechanisms of action. We should have those in mind and also think what else could it do to the immune system and maybe we need to watch people on these agents longer because we know it's not only while the drug is there that it changes something. But there are some modifications that go on for months or years for example.* |
|  | 11 | Biomedical Research | ART use with LRA-only studies: A*t least during the treatment period, antiretrovirals are maintained so there shouldn't be risk of transmission to sexual partners and such.* |
|  | 15 | Community Member | Long-term follow-up of trial participants: I *think just long-term check-in, which I think is being done, to make sure because some of these drugs can also sort of mess with the genome a little bit in terms of gene expression, and you wouldn't want sort of pro-cancer type genes getting switched on. And so, I think that follow-up is ongoing in the studies that have been done.* |
| **Additional Considerations** | | | |
| **Ensuring Improvement Over Standard ART for PLWH** | | | |
| Weigh expected risks against potential benefits | 02 | Biomedical Researcher | *I would think that…, first of all, let's assume that the goal of the intervention is that people will be able to be off antiretroviral therapy for some reasonable period of time, even if not forever… It would have to be long enough to make the time of the intervention and the risk of the intervention worthwhile… If the intervention has a pretty reasonable chance of succeeding at some reasonably acceptable level of toxicity, then, for some people, that may be well worth the investment.* |
|  | 13 | Biomedical Researcher | *You still want to make sure that people have the right balance between side effects and benefits, meaning the potential of a cure probably could balance out somewhat more side effects than with ART… but, if they are serious side effects, I think it would get very difficult to justify since we have such a great comparative of ART that works very well, it's tolerated and gives people a really almost normal life.* |
|  | 01 | Community Member | *I mean, if I have to put up with something inconvenient or suffer in any way, if the payoff is my virus is either eliminated or durably suppressed without meds, then chances are it's going to be worth it.* |
| Different ways to think about improvement | 10 | Biomedical Researcher | *So I do think that there are many ways of thinking about what improvement means from individual's perspective, from a public health perspective, from a cost effectiveness perspective, and all those things need to be considered.* |
| Integrating community perspectives to define meaningful clinical benefit | 08 | Regulator | *It will be complicated and will require a lot of thinking and discussion with the patient community about what they see is a clinically meaningful benefit to them.* |
|  | 01 | Community Member | *So, I think you just have to look at the big picture and that's where having CABs and community advisors comes in, right?* |
| Integrating issues of accessibility, affordability, and stigma for PLWH | 05 | Biomedical Researcher | *I think from a more general perspective you do get into issues of accessibility, affordability, tolerability, and stigma.* |
|  | 18 | Biomedical Researcher | *People on antiretroviral therapy still have HIV, and they might stop their antiretroviral therapy, and they live with both external and internal stigma that impacts their life to an incredible degree.* |
| **Challenges to Combination HIV Cure Regimens** | | | |
| Challenge of collaboration among pharmaceutical companies | 02 | Biomedical Researcher | *Well, there's the practical challenge of getting everybody to agree to put the intervention together. Often that becomes an issue of who has developed or owns each of these interventions, particularly when pharmaceutical companies are involved, or biotech companies. They have to be willing to work with each other, and sometimes that's easier to achieve than in other times. So, that's probably the biggest limitation.* |
|  | 09 | Biomedical Researcher | *I think the other thing is more at the level of people playing in the sandbox, and by people, I mean industry. And so, how do you approach in a real world situation, not in a monkey study but in people, with the possibility of a commercialized product or a treatment regimen of commercialized products, plural, that come from different companies? How do you do that, both regulatory-wise and, "Oh, it's not 50-50; our drug is doing 70% of the work." You get into all that kind of thing.* |
|  | 11 | Biomedical Researcher | *Of course, a hurdle in doing combination studies often is also getting the various companies that own the different therapeutics to work together.* |
|  | 13 | Biomedical Researcher | *Like, for example, if a company supports a trial and they have one drug and the drug you want to combine it with is from another company, that might be a big challenge. It might be hard to get them together.* |
|  | 01 | Community Member | *Certainly, when you get lots of players involved and especially industry, they've got to be willing to share their ideas and concepts with each other.* |
|  | 03 | Community Member | *Well, the problem is this, and I know this question comes later, but it's often what you have available. It's really not what are the best combinations. What's available at the time of your protocol? What drug company is willing to work with another drug company, if they come from different places?* |
| Lack of coordination | 05 | Biomedical Researcher | *I think the best approach to HIV cure-related research and progress is through supporting promising lines of research wherever they are and giving as many resources as you can, which of course is the tricky thing, to people who can explore them. There haven't really been good head to heads yet, and this is where I think it comes to the lack of coordination of combination that is occurring from authorities, let's call them that, to mandate a valid experiment which could be the Manhattan project run in different settings… There should be a head-to-head mouse challenge because combinations can be tested much quicker in mice.* |
| Timing of analytical treatment interruption | 02 | Biomedical Researcher | *When, in the sequence of interventions, should the treatment interruption be done? That really depends very much on the specific intervention. If it involves a therapeutic vaccine, then certainly after you've completed the vaccination series, and then it depends a lot on the pharmacodynamics of the other components of each intervention. When does it make the most sense to interrupt therapy?* |
| Danger of small uncontrolled experiments that may be inconclusive | 02 | Biomedical Researcher | *And that study that [Biomedical Researcher] did with, I think it included maraviroc and, ironically, today it also included hydroxychloroquine as an immune modulator, was done appropriately with control. And, of cours,e the only person who seemed to have a response ended up being a control participant. And that highlighted to me the dangers of doing these smaller uncontrolled experiments.* |
| Clinical relevance for PLWH | 09 | Biomedical Researcher | *The question: is it clinically relevant? Is there a clinical difference for the patient, or have you just put that individual through some cool technology, and it didn't really do anything for them?* |
| Future commercialization potential of combination HIV cure products | 09 | Biomedical Researcher | *And commercialization: how is it promoted, how is it made available, what are the challenges? There may be certain differences in a combination product in the US versus a combination product in Europe, for example, in terms of how it actually gets to the pharmacy, or even how it's packaged.* |
| **Engaging Communities around Combination HIV Cure Research** | | | |
| Managing expectations and clarifying goals of the research | 08 | Regulator | *More likely, it's going to be something short and brief in how do you communicate these complex issues in short communications meant to grab people's attention.* |
|  | 02 | Biomedical Researcher | *And, I think, it's very important that we continue to talk about what the goals of the research are, its importance, and to be able to describe in terms that are understandable to people at various levels of education, and scientific sophistication, and medical sophistication, to understand why they may be asked to participate in studies of this type.* |
|  | 16 | Biomedical Researcher | *So, people really need to understand that this first generations of studies are aimed at just proving proof of concept, getting something across the finish line, because science is quite iterative… How clinical trial research is done, in this iterative process, with a lot of these studies looking for proof of concept and the rules of such studies, that'd be helpful if more people understood that.* |
|  | 17 | Community Member | *From the perspective of the community advocates and activists, having a handle on the risk-benefit and managing expectations for the participants and the broader population.* |
| Research capacity building and sustained dialogue | 10 | Biomedical Researcher | *I think the more of those, the more crosstalk there is between those groups, the better we are able to tailor sort of what is truly desirable in the community with what is feasible and ultimately our goals for cure strategies.* |
| Building trust in HIV cure research | 01 | Community Member | *There's a lot of mistrust [among] people… who aren't part of the research enterprise… And that's a long trust-building process, right?* |
